# A-site cation–pseudohalide co-design for tin halide perovskite transistors

**DOI:** 10.1126/sciadv.aeh2062

**Published:** 2026-10-30

**Authors:** Bum Ho Jeong, Cheong Beom Lee, Hansol Park, Seung Yoon Shin, Soobin Heo, Kyeounghak Kim, Hui Joon Park

**Affiliations:** ^1^ Department of Organic and Nano Engineering, Hanyang University, Seoul 04763, Korea.; ^2^ Human-Tech Convergence Program, Hanyang University, Seoul 04763, Korea.; ^3^ Department of Chemical Engineering, Hanyang University, Seoul 04763, Korea.; ^4^ Department of Semiconductor Engineering, Hanyang University, Seoul 04763, Korea.; ^5^ Institute of Nano Science and Technology, Hanyang University, Seoul 04763, Korea.

## Abstract

Tin halide perovskites are promising lead-free semiconductors for next-generation electronics; however, their implementation is constrained by a fragile crystal framework, facile Sn^2+^ oxidation, and rapid crystallization kinetics. Here, we establish a molecular design principle for non-metallic thiocyanate (SCN^−^) additives that stabilizes the formamidinium tin iodide (FASnI_3_) lattice and enables high-performance, stable p-channel thin-film transistors (TFTs). Density functional theory calculations reveal that the Sn–X–Sn bond strength in the lattice and the halide migration barriers can be tuned by the size of the A-site cation and its hydrogen-bonding capability with SCN^−^ and I^−^. In particular, we find that both the Sn–SCN–Sn bonding interaction and the iodine migration barrier are maximized when guanidinium thiocyanate (GuaSCN) is employed. Consistently, GuaSCN enhances the crystallinity and grain size of tens-of-nanometer-thick FASnI_3_ films, prolongs carrier lifetimes, and suppresses Sn^4+^ formation, without altering the intrinsic bandgap. TFTs incorporating GuaSCN achieve a field-effect mobility above 88 cm^2^ V^−1^ s^−1^, an *I*_on/off_ ratio exceeding 4 × 10^9^, negligible hysteresis, and superior operational and shelf stability across hundreds of devices.

## INTRODUCTION

Metal halide perovskites have emerged as promising next-generation semiconductors for a wide range of optoelectronic devices—including solar cells (*1*), light-emitting diodes (*2*), photodetectors (*3*), and lasers (*4*)—as well as for electronic components such as thin-film transistors (TFTs), owing to their direct bandgaps, long charge-carrier diffusion lengths, high charge-carrier mobility, and compatibility with low-temperature processing (*5*). However, fully translating these intrinsic advantages into high-performance, operationally stable devices requires precise control over film crystallization and rigorous suppression of defect formation.

Thiocyanate (SCN^**−**^)-based additives, representing a prototypical class of pseudohalide anions, have been widely employed in lead (Pb) halide perovskites to regulate crystal growth, enlarge grain size, and passivate defects at surfaces and grain boundaries (*6*–*9*). By contrast, studies in tin (Sn) halide perovskites—highlighted as environmentally benign Pb-free alternatives—remain comparatively limited. This reflects not only the relatively early stage of Sn halide perovskite research, but also the stringent processing constraints imposed by rapid crystallization, the high chemical reactivity of Sn-based precursors, and the facile oxidation of Sn^2+^, which together give rise to high defect densities (*10*, *11*), as well as the greater difficulty in controlling Sn–SCN coordination compared with Pb–SCN. Recent reports focusing on metal-cation SCN sources, such as Sn(SCN)_2_ and Pb(SCN)_2_, show that these additives can promote grain growth, mitigate self p-doping, and reduce interfacial trap density via S/N bidentate chelation to undercoordinated Sn^2+^ centers (*11*–*13*). However, such strong S/N bidentate chelation can severely retard crystallization and leave SCN-rich intermediates, which may introduce residual stress and microdefects within the film, increase long-term ion migration, and diminish photo- and thermal stability (*12*, *13*). Moreover, metal-SCN precursors are moisture-sensitive and risk metallic contamination in Sn-only systems (e.g., Pb carryover from Pb(SCN)_2_) (*10*–*13*).

These limitations have motivated the exploration of non-metallic, SCN-based additives paired with organic cations, which circumvent metal-centered chelation while still enabling controlled tuning of the structural and electronic properties of Sn halide perovskites. In particular, SCN-based salts incorporating different organic cations have been reported to play distinct roles: they can mitigate Sn oxidation and self p-doping, promote more ordered film growth with reduced energetic disorder, and effectively passivate defects in both 3D and 2D/3D Sn halide architectures (*14*–*17*). Taken together, these observations indicate that SCN^**−**^ should be regarded not merely as a generic pseudohalide anion, but as a multidimensional design parameter whose function is governed by the structure, bonding characteristics, and dimensionality of the co-introduced cation.

Here, we introduce a design principle for non-metallic SCN-based additives that efficiently stabilize the crystal structure of the Sn halide perovskite formamidinium tin iodide (FASnI_3_). We show that the size and hydrogen-bonding capability of the organic cation govern the configuration of the pseudohalide SCN^**−**^, thereby modulating the Sn–X–Sn bond strength and the robustness of the inorganic framework. By incorporating guanidinium thiocyanate (GuaSCN)—identified through this A-site–pseudohalide co-design strategy—into tens-of-nanometer-thick FASnI_3_ films, where device performance is particularly sensitive to lattice stability, we achieve high-performance, durable TFT operation.

In Sn halide perovskite TFTs, the facile oxidation of Sn^2+^ to Sn^4+^ (*18*), which generates deep defects and induces strong self p-doping (*19*), complicates channel depletion and hampers efficient switching (*20*). In addition, the intrinsically rapid crystallization kinetics of Sn-based perovskites often lead to non-uniform films with small grains and defect-rich grain boundaries, resulting in pronounced hysteresis during transistor operation (*21*). Recent studies on tin halide perovskite TFTs have demonstrated impressive progress in high-mobility p-channel operation through A-site cation engineering, crystallization control, and vapor-deposition-based film formation (*22*–*24*). Nevertheless, simultaneously achieving high mobility, suppressed hysteresis, and long-term operational stability remains challenging because charge transport in Sn halide perovskites is strongly coupled with defect formation and ion migration. In this work, the optimized FASnI_3_ TFTs incorporating GuaSCN exhibit a remarkable field-effect mobility (μ_FE_ > 88 cm^2^ V^**−**1^ s^**−**1^), a high on/off current ratio (*I*_on/off_ > 4.0 × 10^9^), and strongly suppressed hysteresis, while maintaining excellent operational stability. These findings not only provide mechanistic insight into the role of SCN-based additives in stabilizing Sn halide perovskites at the atomic scale, but also open up new avenues for the design of high-performance, stable, and reproducible p-channel TFTs, which still lag behind state-of-the-art n-type semiconductors.

## RESULTS

### Molecular design of non-metallic thiocyanate-based additives

We first examine how the local structural environment of FASnI_3_ evolves in the absence and presence of SCN^−^ as a function of the A-site cation—cesium (Cs^+^), ammonium (NH_4_^+^), methylammonium (MA^+^), formamidinium (FA^+^), dimethylammonium (DMA^+^), ethylammonium (EA^+^), guanidinium (Gua^+^), acetamidinium (AA^+^), and trimethylammonium (TMA^+^)—using density functional theory (DFT) calculations (Fig. 1, A to C and fig. S1). In this analysis, we focus on two key descriptors: the effective size of the A-site cation (Fig. 1, A and B) and the resulting Sn–X–Sn bond strength within the crystal (Fig. 1C). Because it is difficult to define meaningful ionic radii for asymmetric molecular cations, we instead estimated their spatial occupation by evaluating the volume of the cuboctahedral cavity formed by the 12 surrounding anions (Fig. 1B). Overall, cations with fewer atoms tend to yield smaller cuboctahedral volumes. For reference, for small cations such as Cs^+^, NH_4_^+^, and MA^+^, the cavity volume noticeably differs between I^−^ and SCN^−^. In particular, NH_4_^+^ and MA^+^ lead to smaller cavity volumes when I^−^ is replaced by SCN^−^, which we attribute to hydrogen bonding between NH_4_^+^/MA^+^ and SCN^−^ (discussed in more detail below). By contrast, incorporation of Cs^+^ increases the cavity volume upon SCN^−^ substitution, likely due to the absence of hydrogen bonding. These observations suggest that hydrogen bonding between the A-site cation and SCN^−^ is a critical factor in shaping the crystal lattice and, consequently, the Sn–X–Sn bonding environment.

**Fig. 1.**
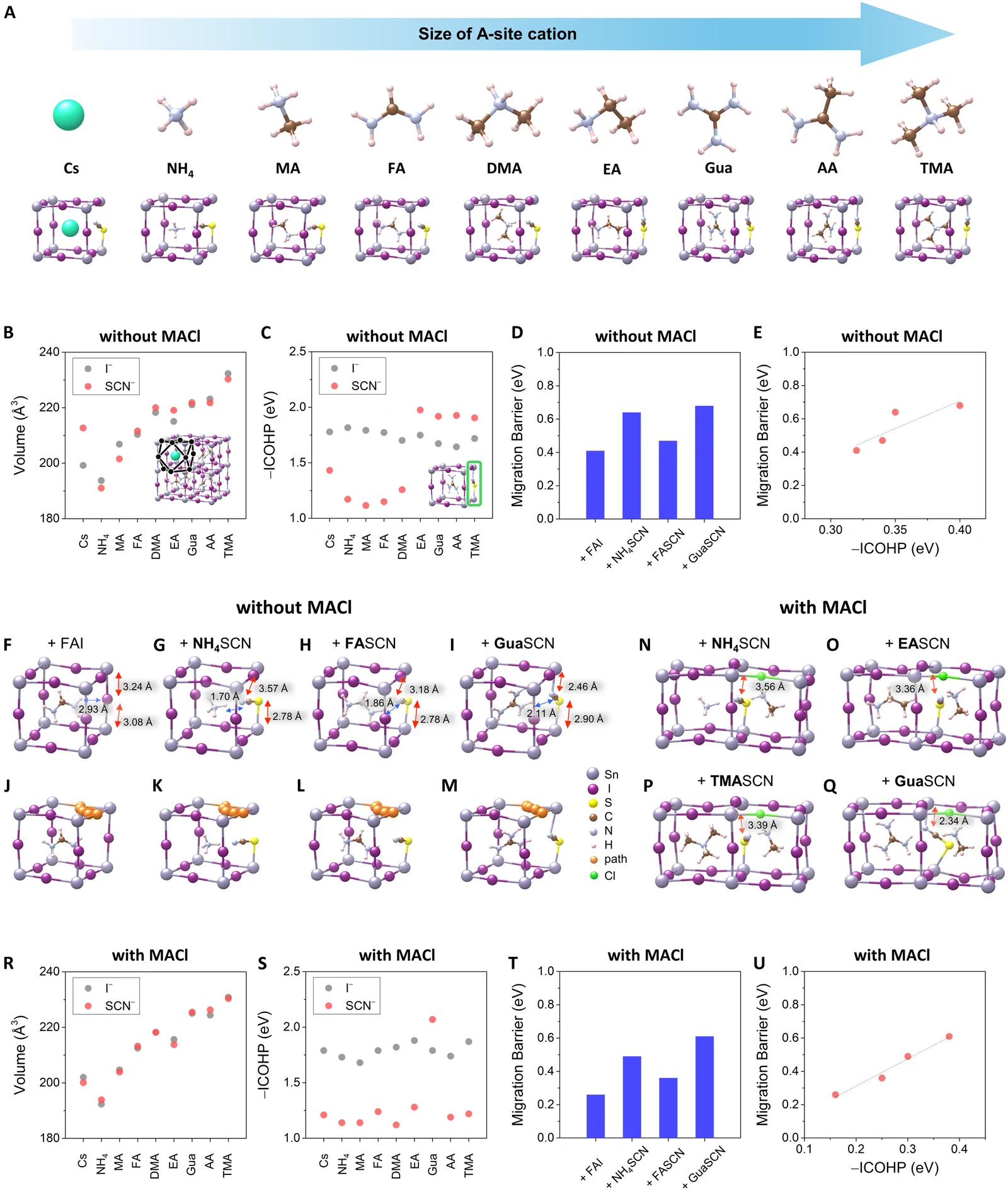
Atomistic design of A-site–SCN coupling in FASnI_3_ by DFT. (**A**) Relative sizes of A-site cations (top) and the corresponding local environments in optimized FASnI_3_ structures with SCN incorporation (bottom) (without MACl). (**B**) Volume of the cuboctahedral cavity with different A-site cations (without MACl). Black lines schematically outline the cavity formed by the surrounding iodide ions. (**C**) Sum of the −ICOHP values for the framework bonds (Sn-N + S-Sn), highlighted as a green box, for different A-site cations (without MACl). (**D**) Iodide migration barriers for the systems shown in [(J) to (M)] (without MACl). (**E**) Correlation between the −ICOHP of NH···I interaction at transition state and the corresponding migration barrier (without MACl). Local environments of the optimized (**F**) pristine FASnI_3_ and FASnI_3_ containing (**G**) NH_4_SCN, (**H**) FASCN, and (**I**) GuaSCN (without MACl). Red arrows indicate the bond distances between I or SCN and Sn, whereas blue arrows indicate NH···I or NH···N distances associated with hydrogen bonding. Iodide migration pathways in (**J**) pristine FASnI_3_ and FASnI_3_ containing (**K**) NH_4_SCN, (**L**) FASCN, and (**M**) GuaSCN, with the migrating iodide highlighted in orange (without MACl). Local environments of the optimized FASnI_3_ containing (**N**) NH_4_SCN, (**O**) EASCN, (**P**) TMASCN and (**Q**) GuaSCN (with MACl, FA_0.875_MA_0.125_SnI_2.85_Cl_0.15_). Red arrows indicate the bond distance between SCN and Sn. (**R**) Volume of the cuboctahedral cavity for different A-site cations (with MACl, FA_0.875_MA_0.125_SnI_2.85_Cl_0.15_). (**S**) Sum of the −ICOHP values for the framework bonds (Sn-N + S-Sn) for different A-site cations (with MACl, FA_0.875_MA_0.125_SnI_2.85_Cl_0.15_). (**T**) Iodide migration barrier (with MACl, FA_0.875_MA_0.125_SnI_2.85_Cl_0.15_). (**U**) Correlation between the −ICOHP of NH···I interaction at transition state and the corresponding migration barrier (with MACl, FA_0.875_MA_0.125_SnI_2.85_Cl_0.15_).

The corresponding Sn–X–Sn bond strength in FASnI_3_ with different SCN-based additives was evaluated using the crystal orbital Hamilton population (COHP) method. For I^−^, the Sn–I–Sn bond strength, represented by the integrated −COHP (−ICOHP) value, shows little dependence on the A-site cation (Fig. 1C, grey). In contrast, upon replacing I^−^ with SCN^−^, the bond strength becomes more sensitive to the choice of A-site identity. Systems incorporating Cs^+^, NH_4_^+^, MA^+^, FA^+^, or DMA^+^ exhibit relatively weaker Sn–SCN–Sn interactions than those containing EA^+^, Gua^+^, AA^+^, or TMA^+^ (Fig. 1C, red). Together with the cavity-volume analysis, these results indicate that small A-site cations can destabilize the inorganic framework, whereas larger cations are generally preferred for forming stronger Sn–X–Sn bonds in the inorganic network, provided that excessively bulky cations—which can induce framework distortion and trigger phase transitions—are avoided (*25*).

To clarify in more detail how the A-site cation governs SCN^−^ coordination and the corresponding Sn–SCN–Sn interaction, we selected two SCN-based salts, NH_4_SCN and GuaSCN, which lie in distinct regimes of bond strength (Fig. 1C), together with FASCN as a benchmark. We then analyzed how these SCN-based additives with different cations modify the local environment and ion-migration behavior within the perovskite lattice. The incorporation of different A-site cations (A = NH_4_, FA, or Gua) leads to distinct modifications in the local structure of the perovskite (Fig. 1, F to I). In particular, the configuration of SCN^−^ is strongly influenced by the A-site cation, and the N atom of SCN^−^ tends to be drawn toward the H atoms of the A-site organic cations via hydrogen bonding. For NH_4_ incorporation (NH_4_SCN), the N atom of SCN^−^ is oriented toward NH_4_ rather than towards the Sn center of the inorganic framework, with a Sn–N distance of 3.57 Å (Fig. 1G), indicating that the interaction between SCN^−^ and Sn is primarily mediated through the S atom. A similar trend is observed in FASCN (Sn–N distance of 3.18 Å, Fig. 1H). By contrast, a significantly shorter Sn–N distance of 2.46 Å is obtained when Gua occupies the A-site (Fig. 1I), implying that both N and S atoms form chemical bonds with Sn in the GuaSCN system. Consequently, the configuration of the pseudohalide SCN^−^ is strongly modified by the identity of the A-site cation.

Because ion migration is a major origin of the poor stability of halide perovskites (*26*), we next evaluated the effect of an iodine vacancy on I^−^ migration through the perovskite lattice (Fig. 1, J to M). The DFT-calculated migration barriers are 0.41 eV, 0.64 eV, 0.47 eV, and 0.68 eV for pristine FASnI_3_, NH_4_SCN-, FASCN-, and GuaSCN-containing systems, respectively (Fig. 1D). To gain a fundamental understanding of the different I^−^ migration barriers induced by different A-site cations in the SCN additives, we analyzed the interaction between the migrating I^−^ and the H atoms of the A-site cation using the COHP method, since hydrogen bonding between I^−^ and A-site cation can hinder halide migration (*27*). The −ICOHP values for the H···I interaction are 0.32 eV, 0.35 eV, 0.34 eV, and 0.40 eV for pristine FASnI_3_, NH_4_SCN, FASCN, and GuaSCN, respectively (Fig. 1E). These results reveal that the strength of hydrogen bonding correlates with the I^−^ migration barrier, indicating that stronger H···I interactions more effectively suppress halide migration.

However, it is worth noting that the iodide migration barrier cannot be explained solely by the average hydrogen-bonding strength and the steric size. Although FA^+^ exhibits an interaction strength (−ICOHP = 0.34 eV) comparable to that of NH_4_^+^ (0.35 eV) and is larger in size, the NH_4_^+^-containing system shows a migration barrier of 0.64 eV, which is much closer to that of the GuaSCN-containing system (0.68 eV) than to that of the FA^+^-containing system only (0.47 eV). This indicates that the migration barrier is governed not only by steric effects, but also by the local coordination environment and the spatial distribution of interactions within the lattice (*28*, *29*). Owing to its compact and highly symmetric structure, NH_4_^+^ can provide a more isotropic local interaction environment that more uniformly constrains iodide migration pathways, whereas the more anisotropic geometry of FA^+^ may leave locally less constrained diffusion pathways. Such differences in local structural symmetry and coordination environment are therefore expected to affect lattice stabilization and the suppression of halide migration (*25*). This interpretation is supported by our DFT results. Molecular cations such as NH_4_^+^ and Gua^+^ provide a more uniform distribution of hydrogen-bonding sites along the migration pathway than FA^+^. Whereas FA^+^ often leaves iodide-transport regions with relatively sparse C–H interactions, NH_4_^+^ and Gua^+^ more effectively anchor the migrating iodide through dense, quasi-isotropic hydrogen bonding. Consistently, our DFT calculations reveal a correlation between the migration barrier and the NH···I distance (fig. S2), highlighting the importance of localized hydrogen-bonding density in stabilizing the inorganic framework. For reference, this calculation is based on the composition FA_0.875_MA_0.125_SnI_2.85_Cl_0.15_; the rationale for this composition is explained in the following section. Overall, these results indicate that incorporation of GuaSCN into FASnI_3_ can enhance device stability not only by strengthening Sn–X–Sn bonding, but also by increasing the I^−^ migration barrier through more effective hydrogen-bond-mediated lattice stabilization.

In the following section, we experimentally evaluate the impact of SCN-based additives on FASnI_3_ films using three representative cases: pristine FASnI_3_, and NH_4_SCN-treated FASnI_3_, and GuaSCN-treated FASnI_3_. In all three cases, 15 mol% methylammonium chloride (MACl), known to enhance the crystallinity of FASnI_3_ (*30*), was added as a common co-additive. Unless otherwise noted, the concentration of the SCN-based additives was fixed at 2.5 mol%, which yielded the optimum film quality. Since these experimental films contain both FA-rich regions and MACl-containing regions, we additionally performed DFT calculations using the actual composition of FA_0.875_MA_0.125_SnI_2.85_Cl_0.15_ (Fig. 1, N to Q and R to U and fig. S3, C and D). Due to the finite size of the DFT supercell, the composition was approximated as FA_0.875_MA_0.125_SnI_2.85_Cl_0.15_, which is the closest representation to the experimental composition (FA_0.85_MA_0.15_SnI_2.85_Cl_0.15_). In this model, the cuboctahedral volume increased upon cation substitution (Fig. 1R), consistent with the trend observed for pure FASnI_3_ (Fig. 1B). Similarly, the corresponding iodide migration barriers (Fig. 1, T and U) closely followed the trends obtained for pure FASnI_3_ (Fig. 1, D and E). Interestingly, in the presence of MACl, larger cations such as EA, AA, and TMA often fail to maintain stable hydrogen-bonding networks with SCN^−^, leading to competitive interactions in which the N atom of SCN^−^ preferentially coordinates with neighboring MA cations (Fig. 1, N to Q). This behavior substantially weakens the Sn–SCN–Sn linkage. In contrast, Gua uniquely preserves a robust Sn–SCN–Sn framework even in the presence of MA (Fig. 1, Q and S). The Sn–N distances in the EA, TMA and Gua cases are3.36, 3.39, and 2.34 Å, respectively. This enhanced stability arises from the synergistic combination of its large A-site volume and strong hydrogen-bonding capability, which effectively anchors the SCN configuration against external perturbations. This is consistent with the results obtained for the pure FASnI_3_ model, in which Gua likewise maintains a strong Sn–SCN–Sn linkage (Fig. 1, C and I). Accordingly, Gua was identified as the most suitable candidate for achieving long-term structural stability even in the FA_0.875_MA_0.125_SnI_2.85_Cl_0.15_ system. NH_4_ and Gua were therefore selected as representative candidates for further experimental investigation, because both form strong hydrogen bonds with SCN^−^ while exhibiting a large difference in A-site volume. By contrast, MA, EA, AA, and TMA possess either more anisotropic geometries or weaker hydrogen-bonding interactions. NH_4_ and Gua thus provide a simple yet informative model system for examining the respective roles of A-site volume and hydrogen bonding in stabilizing the Sn–SCN–Sn framework.

### Structural and optoelectronic improvements induced by SCN-based additives

The structural impact of SCN-based additives on FASnI_3_ films was experimentally investigated using X-ray diffraction (XRD). As noted above, in all three cases—pristine FASnI_3_ and FASnI_3_ films containing NH_4_SCN or GuaSCN—15 mol% MACl was commonly added, and the concentration of the SCN-based additives was fixed at 2.5 mol%. All three samples retain the characteristic reflections of a 3D cubic perovskite structure with the Pm-3 m space group (*30*, *31*), confirming that neither NH_4_SCN nor GuaSCN disrupts the underlying lattice symmetry (Fig. 2A). However, noticeable differences arise in peak sharpness and intensity. Both SCN-treated films exhibit enhanced crystallinity compared with the FASnI_3_ without SCN, as evidenced by reduced peak broadening. A magnified view of the (200) reflection (Fig. 2B) highlights this trend: the NH_4_SCN-treated film shows a moderate narrowing of the full width at half maximum (FWHM), whereas the GuaSCN-treated film exhibits the most pronounced peak sharpening. This progressive improvement—from pristine FASnI_3_ to NH_4_SCN to GuaSCN—indicates that SCN-based additives promote more ordered crystallization, with GuaSCN most effectively enhancing long-range structural coherence (*32*). Although the (200) reflection is highlighted because of its strong intensity and well-defined peak shape, the (100) reflection shows the same trend, with progressive peak narrowing from pristine FASnI_3_ to NH_4_SCN-treated and GuaSCN-treated FASnI_3_ films (fig. S4). No meaningful peak shift is observed for either the (100) or (200) reflection within the resolution of the XRD measurement, indicating that the average lattice parameter is largely preserved after SCN-based additive incorporation. Therefore, the primary effect of the additives is not substantial lattice expansion or contraction but improved crystallographic order. This improvement is attributed to hydrogen-bond-mediated modification of the local SCN^−^ coordination environment, which strengthens the Sn–X–Sn framework, reduces local structural disorder and microstrain, and thereby leads to sharper diffraction peaks.

**Fig. 2.**
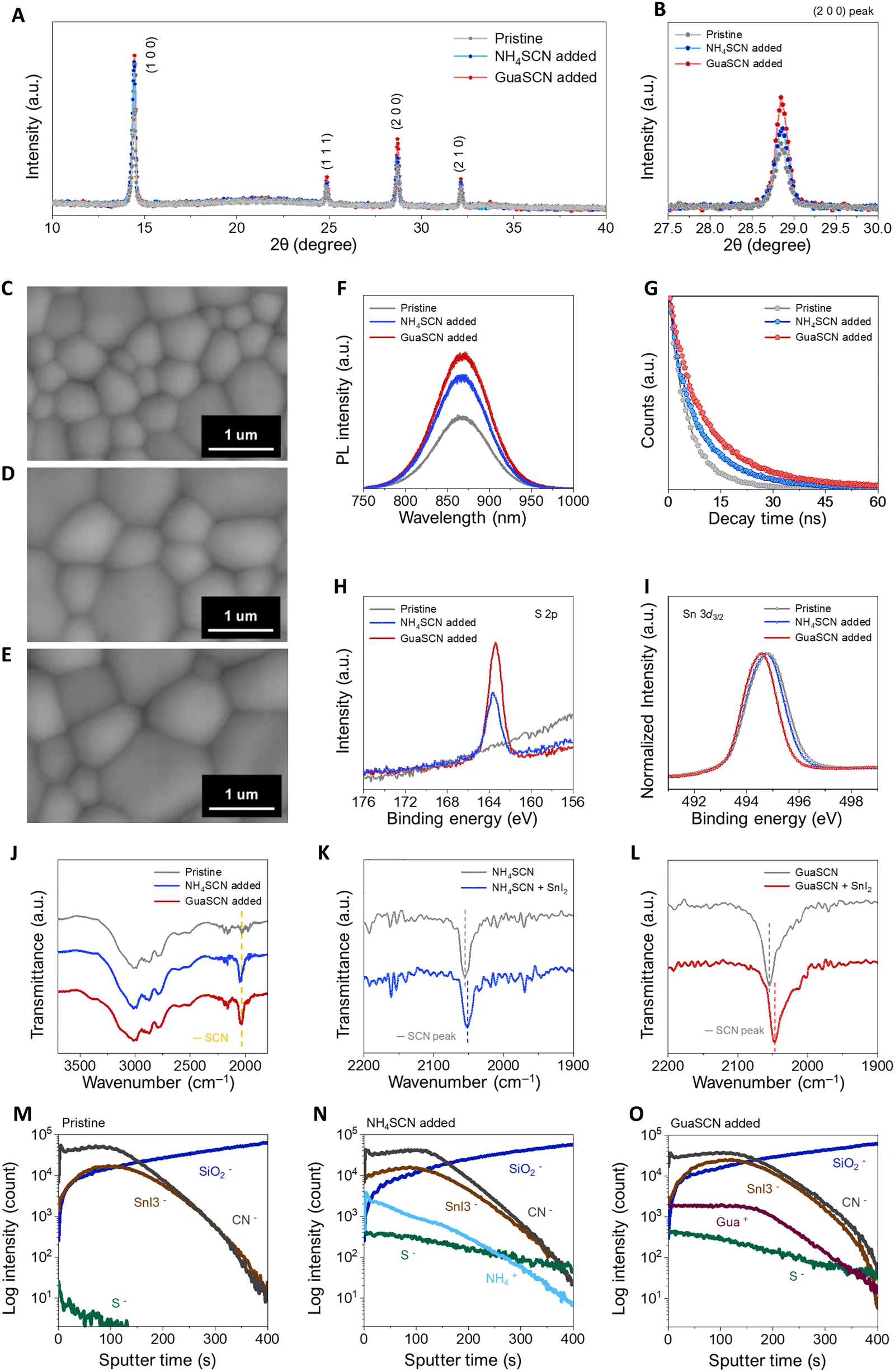
A-site-cation-coupled stabilization of FASnI_3_ by SCN-based additives. (**A**) XRD patterns of FASnI_3_, NH_4_SCN-treated, and GuaSCN-treated FASnI_3_ films. (**B**) Enlarged view of the (200) reflection from (A). SEM images of (**C**) FASnI_3_, (**D**) NH4SCN-treated FASnI_3_, and (**E**) GuaSCN-treated FASnI_3_ films. (**F**) Steady-state PL spectra of FASnI_3_, NH_4_SCN-treated, and GuaSCN-treated FASnI_3_ films. (**G**) Time-resolved PL decay curves for the same series of films. High-resolution XPS core-level spectra of (**H**) S 2p and (**I**) Sn 3d for FASnI_3_, NH_4_SCN-treated, and GuaSCN-treated FASnI_3_ films. FT-IR spectra probing the *v*(C ≡ N) vibration in (**J**) perovskite films and binary SnI_2_-SCN mixtures, specifically (**K**) NH_4_SCN versus SnI_2_-NH_4_SCN and (**L**) GuaSCN versus SnI_2_-GuaSCN. ToF-SIMS depth profiling analysis of SCN-based additive-treated FASnI_3_ films. (**M**) pristine FASnI_3_, (**N**) NH_4_SCN-treated FASnI_3_, and (**O**) GuaSCN-treated FASnI_3_ films.

Steady-state photoluminescence (PL) measurements (Fig. 2F) further reveal a substantial enhancement in emission intensity upon incorporation of SCN-based additives, consistent with improved crystal quality and reduced defect densities that suppress nonradiative recombination (*33*). Relative to the FASnI_3_ film without SCN, the NH_4_SCN-treated film exhibits increased radiative emission, while the GuaSCN-treated film shows the strongest PL signal. Time-resolved PL (TRPL) measurements provide additional quantitative insight (Fig. 2G). The decay curves were fitted with a biexponential model, yielding fast (τ_1_) and slow (τ_2_) components that are typically attributed to trap-assisted nonradiative recombination and band-to-band radiative recombination, respectively (*34*). The average carrier lifetime (τ_avg_) increases from 7.86 ns for FASnI_3_ without SCN to 11.57 ns for the NH_4_SCN-treated film, and further to 16.25 ns for the GuaSCN-treated film, confirming that SCN-based additives—most notably GuaSCN—effectively reduce recombination pathways. Detailed fitting parameters are summarized in table S1.

Scanning electron microscopy (SEM) further elucidates the morphological evolution induced by SCN-additives (Fig. 2, C to E). Compared with the FASnI_3_ film without SCN, both NH_4_SCN and GuaSCN treatments yield larger, more uniformly connected grains with improved surface coverage, indicative of retarded nucleation and more complete grain coalescence with the most pronounced grain enlargement observed for GuaSCN. To confirm that this grain enlargement is not limited to a local region, we additionally acquired low-magnification SEM images over larger film areas (fig. S5). The GuaSCN-treated film exhibits uniformly enlarged and well-connected grains across the extended field of view, without obvious localized aggregation or discontinuous regions, confirming that the grain coarsening observed in the high-magnification SEM images is representative of the overall film morphology. The progression in film morphology—from pristine FASnI_3_ to NH_4_SCN- and then to GuaSCN-treated films—parallels the corresponding improvements in crystallographic order and photophysical properties observed in the XRD and PL measurements.

The observed improvements in film quality induced by the different SCN-based additives support the A-site–pseudohalide coupling revealed by DFT calculations. SCN-based additives have previously been reported to modulate perovskite crystallization, promote grain growth, and induce large-grain formation through crystallization control and Ostwald-ripening-related processes (*9*, *32*). In our system, however, the extent of grain growth strongly depends on the identity of the A-site cation. NH_4_SCN provides only moderate improvement, whereas GuaSCN leads to much more pronounced grain enlargement, indicating that the crystallization behavior is governed not by SCN^−^ alone, but by its coupling with the A-site cation. The stronger hydrogen-bonding interaction of Gua^+^ reorients SCN^−^ toward the inorganic framework, strengthens the Sn–X–Sn bonding network, and reduces local structural disorder. Such lattice stabilization is expected to suppress micro-strain accumulation and vacancy-mediated disorder, both of which are known to influence defect formation and ion migration in halide perovskites (*28*, *35*).

Beyond conventional crystallization modulation, the suppression of Sn-vacancy formation by different SCN-based additives was further evaluated by DFT calculations. As noted earlier, whereas the Sn–I–Sn network is relatively insensitive to the A-site cation, the Sn–SCN–Sn linkage is highly sensitive to the A-site identity. In particular, for smaller cations such as NH_4_^+^, competitive hydrogen bonding between the A-site cation and SCN^−^ weakens the Sn–N coordination (top panel in fig. S6C), as reflected by the low −ICOHP value (0.03 eV; fig. S6B). This weakening lowers the formation energy of Sn vacancies, which are closely associated with the poor stability of Sn halide perovskites. By contrast, Gua effectively suppresses Sn-vacancy formation by stabilizing the Sn–SCN–Sn network (bottom panel in fig. S6C), as evidenced by the much larger −ICOHP value (1.30 eV; fig. S6B). These results suggest that the improved film morphology originates not only from enhanced crystallization, but also from the concurrent suppression of defect formation.

We further calculated the vacancy formation energies (*E*_vf_) of both iodide and Sn vacancies for different A-site cations (fig. S7). In all cases considered, iodide-vacancy formation (fig. S7A) is less favorable than Sn-vacancy formation (fig. S7B). Moreover, the Sn atom coordinated to the N side of SCN^−^ (top panel in fig. S7B) exhibits a lower vacancy formation energy than the Sn atom bonded to the S side (bottom panel in fig. S7B), indicating that the Sn–S bond is stronger than the Sn–N bond. These results confirm that A-site-dependent hydrogen-bonding interactions modulate the local SCN configuration and, in turn, strongly influence Sn-vacancy formation, particularly through their effect on Sn–N coordination.

Meanwhile, optical absorption measurements analyzed using Tauc plots confirm that the SCN-based additives do not significantly alter the intrinsic bandgap of FASnI_3_, with all films exhibiting nearly identical direct-bandgap onsets (fig. S8B) (*36*). The corresponding UV–vis spectra are provided in fig. S8A, and their minimal variation indicates that the additives do not appreciably perturb the fundamental electronic structure. We attribute this to the relatively low concentration of SCN-based additives, which is insufficient to significantly modify the overall dimensionality or electronic band structure of the perovskite lattice.

### Chemical interactions between SCN-based additives and Sn halide perovskites

The enhanced crystallization afforded by the SCN-based additives is closely associated with their stronger interaction with the perovskite framework. This aspect was probed by Fourier-transform infrared spectroscopy (FT-IR) (Fig. 2, J to L). In the additive-treated films, a distinct absorption band appears in the ν(C ≡ N) stretching region (∼2060–2100 cm^−1^) (*37*), whereas no corresponding feature is observed in the pristine film (Fig. 2J). Particularly, the ν(C ≡ N) band of the GuaSCN-treated film is shifted to slightly lower wavenumber than that of the NH_4_SCN-treated film, indicating that the C ≡ N unit experiences a more strongly perturbed vibrational environment—and hence a stronger interaction with the perovskite framework—when Gua^+^ is present. The interactions between SCN^−^ and Sn^2+^ in the presence of different cations were further examined by characterizing SnI_2_–SCN mixture solutions (Fig. 2, K and L). The SnI_2_-NH_4_SCN mixture exhibits a ν(C ≡ N) band only marginally red-shifted relative to that of the neat NH_4_SCN additive, indicating relatively weak interaction between SCN^−^ and Sn^2+^ in the presence of NH_4_^+^. In contrast, the SnI_2_–GuaSCN mixture shows a much more pronounced red-shift, demonstrating that the SCN–Sn interaction is significantly strengthened when Gua^+^ is present.

The spatial distribution of SCN-based additive-derived species within the perovskite films—pristine, NH_4_SCN-treated, and GuaSCN-treated films—was investigated by ToF-SIMS depth profiling (Fig. 2, M to O). In the GuaSCN-treated film, clear Gua-related and S-related signals are observed throughout the film depth together with the perovskite matrix signals, indicating that GuaSCN-derived species are distributed across the perovskite layer rather than being confined to the surface region. A similar depth distribution of additive-derived species is also observed in the NH_4_SCN-treated film. These results support the view that GuaSCN-derived species interact with the local perovskite framework, consistent with the physical relevance of the A-site–pseudohalide coupling model used in the DFT calculations.

The incorporation of SCN-based additives into the Sn halide perovskite is further confirmed by x–ray photoelectron spectroscopy (XPS). Figure 2H shows distinct S 2p features, absent in the pristine film, confirming the incorporation of SCN^−^ into the perovskite layer. The GuaSCN-added film presents a sharper and more intense S 2p peak, indicating more efficient incorporation of SCN^−^ than in the NH_4_SCN-added counterpart. The stronger and sharper S 2p signal in the GuaSCN-treated film, together with the ToF-SIMS depth-profiling results (Fig. 2, M to O), indicates stronger retention of SCN-derived species and their more intimate interaction with the perovskite framework. In particular, the depth-distributed Gua-related and S-related signals observed throughout the GuaSCN-treated film confirm that GuaSCN-derived species are not confined to the film surface, but are distributed across the perovskite layer. Complementary changes are observed in the Sn 3d spectra (Fig. 2I and fig. S9). Compared with the pristine film, which exhibits a broad Sn 3d_3/2_ peak with a pronounced high-binding-energy shoulder, both SCN-added samples show noticeably sharper Sn 3d peaks with substantially diminished high-binding-energy tails and a slight shift toward lower binding energy, reflecting suppression of Sn oxidation (Sn^4+^ formation) and improved stabilization of Sn^2+^. Quantitative deconvolution of the Sn 3d doublet (fig. S9) reinforces this trend: the Sn^2+^:Sn^4+^ ratio increases from 89.8:5.6 in the pristine film to 92.0:5.3 with NH_4_SCN and further to 93.1:4.7 with GuaSCN, accompanied by a marked reduction in high-binding-energy components associated with oxidized Sn species (*38*). These coordinated changes in peak sharpness, binding-energy position, and oxidation-state distribution collectively demonstrate that SCN-based additives—particularly GuaSCN—effectively reduce Sn-related defects and stabilize the Sn^2+^ framework.

### Performance and operational stability of thin-film transistors

The Sn halide perovskite thin films with crystal structures enhanced by SCN-based additives were employed as the active channels in TFT devices. We fabricated bottom-gate, top-contact TFTs using pristine, NH_4_SCN-treated, and GuaSCN-treated FASnI_3_ channels deposited on a 300-nm-thick thermally grown SiO_2_ dielectric on a heavily doped p-type silicon (p^+^-Si) substrate. Unless otherwise stated, all electrical characterizations were performed in ambient air and in the dark after glass encapsulation. Additional details on device fabrication and the glass-encapsulation procedure are provided in the Materials and Methods section.

Figure 3, A and B presents representative transfer characteristics of TFTs incorporating NH_4_SCN- and GuaSCN-treated FASnI_3_, respectively, measured at *V*_DS_ = −40 V in the saturation regime. The semi-logarithmic |*I*_DS_|–*V*_GS_ characteristics are shown for both forward (off-to-on) and reverse (on-to-off) sweeps, where *I*_DS_ and *V*_GS_ denote the drain current and gate voltage, respectively. For comparison, the transfer characteristics of a TFT based on pristine FASnI_3_ are shown in fig. S10A. The corresponding output characteristics for all three cases exhibit linear behavior at low drain voltages followed by clear current saturation, as shown in Fig. 3, D and E and fig. S10B.

**Fig. 3.**
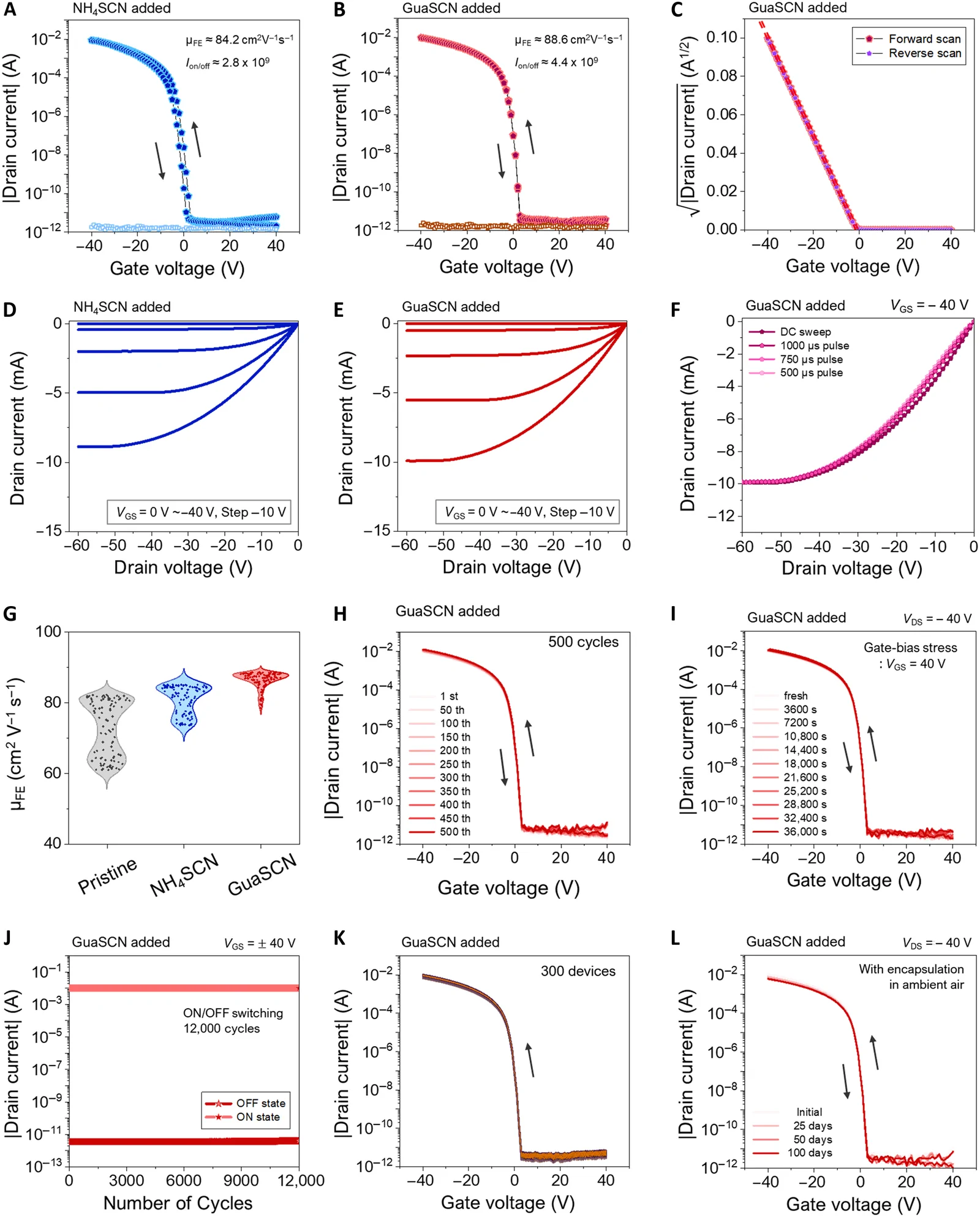
Electrical characteristics and operational stability of SCN-additive-incorporated FASnI_3_ TFTs. Dual-scan transfer characteristics of (**A**) NH_4_SCN-treated and (**B**) GuaSCN-treated TFTs measured at *V*_DS_ = −40 V. The corresponding gate currents (*I*_G_) are also plotted on a logarithmic scale. (**C**) Linear-scale plot of $\sqrt{|I_{\text{DS}}|}$–*V*_GS_ characteristics of the GuaSCN-treated TFT. Output characteristics of (**D**) NH_4_SCN-treated and (**E**) GuaSCN-treated TFTs. (**F**) Output characteristics of the GuaSCN-treated TFT measured at *V*_GS_ = −40 V using conventional dc sweeping and drain-voltage pulses with widths of 1000, 750, and 500 μs. (**G**) Violin plots showing the statistical distributions of μ_FE_ for pristine, NH_4_SCN-treated, and GuaSCN-treated TFTs. Individual data points represent mobility values obtained from 100 devices for each condition (table S3). (**H**) Cycling stability of GuaSCN-treated TFTs over 500 consecutive forward–reverse transfer sweeps (*V*_DS_ = −40 V). (**I**) Dual-scan transfer characteristics of the GuaSCN-treated TFT measured during bias-stress testing under a constant gate bias of 40 V for up to 36,000 s (*V*_DS_ = −40 V). (**J**) Long-term on/off switching endurance of the GuaSCN-treated TFTs under alternating gate pulses (*V*_GS_ = ±40 V, pulse width = 1 s, *V*_DS_ = −40 V) for 12,000 cycles. (**K**) Forward-scan transfer characteristics of 300 GuaSCN-treated TFTs measured at *V*_DS_ = −40 V. (**L**) Shelf-life stability of glass-encapsulated GuaSCN-treated TFTs stored in ambient air for 100 days, evaluated from dual-scan transfer characteristics measured at *V*_DS_ = −40 V.

The GuaSCN-incorporated TFTs exhibit superior device characteristics, with an average μ_FE_ of 86.51 ± 2.23 cm^2^ V^−1^ s^−1^ and an average *I*_on/off_ of (4.20 ± 0.16) × 10^9^. The best-performing GuaSCN device achieves μ_FE_ = 88.6 cm^2^ V^−1^ s^−1^ and *I*_on/off_ = 4.40 × 10^9^, with a threshold voltage (*V*_T_) close to 0 V, surpassing the performance of previously reported Sn halide perovskite TFTs (table S2). To clarify the mobility-extraction procedure, Fig. 3C and fig. S11A shows the $\sqrt{|I_{\text{DS}}|}$–*V*_GS_ characteristics of the GuaSCN-treated TFT on a linear scale. The excellent linearity over a broad gate-voltage range yields an approximately constant derivative, $\partial \sqrt{|I_{\text{DS}}|}/\partial V_{\text{GS}}$, as shown in fig. S11B, demonstrating that the mobility extraction does not rely on a localized steep-slope region of the transfer characteristic. Specifically, the derivative values obtained from the forward transfer sweep over the range −40 ≤ *V*_GS_ ≤ -5 V (yellow-shaded region in fig. S11B) were converted into gate-voltage-dependent μ_FE_ values using the conventional saturation-regime TFT model (fig. S11C). The resulting μ_FE_ values within this gate-voltage range were then averaged to determine the reported μ_FE_ for each device. The same extraction procedure was consistently applied to all devices included in the statistical analysis (table S3). Further details of the mobility-extraction procedure are provided in note S1. The NH_4_SCN-treated devices show slightly lower, but still improved, performance compared with the GuaSCN-treated devices, with an average μ_FE_ of 80.77 ± 4.11 cm^2^ V^−1^ s^−1^ and an average *I*_on/off_ of (2.75 ± 0.27) × 10^9^. In comparison, the pristine FASnI_3_ devices exhibit an average μ_FE_ of 73.01 ± 8.03 cm^2^ V^−1^ s^−1^ and an average *I*_on/off_ of (1.02 ± 0.16) × 10^9^. The statistical analysis was performed using data obtained from 100 devices, and the results are summarized in table S3. The distributions of the individual device data are presented as violin plots in Fig. 3G, revealing a progressive increase in μ_FE_ from pristine to NH_4_SCN- and GuaSCN-treated devices. Unless otherwise noted, all reported device parameters were extracted from the forward transfer sweeps.

Although the GuaSCN-treated films exhibit substantially enlarged grains, the corresponding increase in μ_FE_ is relatively moderate, indicating that charge transport is not governed solely by grain-boundary density. To clarify this point, we additionally evaluated the contact resistance using the transmission line method (fig. S12) (*39*). The extracted contact resistance values indicate that contact-related transport limitation remains non-negligible even after significant grain coarsening, which may originate from the increased surface roughness associated with enlarged grains (*30*). Therefore, while grain enlargement reduces grain-boundary-related disorder, the overall transistor performance is still influenced by additional transport bottlenecks such as residual contact resistance and intra-grain disorder. Importantly, the enlarged grains also contribute to markedly improved subthreshold swings, suppressed hysteresis, enhanced operational stability, and improved device-to-device uniformity, indicating stabilization of both the electronic and ionic defect landscape, which will be discussed later.

Our DFT analysis also indicates that the incorporation of GuaSCN induces an increase in the hole effective mass from 0.18 to 0.22 (fig. S13). Since carrier mobility is inversely proportional to the effective mass, this intrinsic modification partially offsets the benefits gained from reduced grain boundary. Thus, the observed mobility gain might be due to a balanced result of suppressed ion migration and structural stabilization against the slightly increased charge carrier effective mass.

To verify that the extracted mobilities faithfully represent the underlying transport behavior—rather than artifacts arising from non-ideal channel formation—we evaluated the reliability of μ_FE_ using the measurement reliability factor (*r*) (*40*). For the champion GuaSCN-treated TFT with μ_FE_ = 88.6 cm^2^ V^−1^ s^−1^, *r* was 0.971, corresponding to a reliability-corrected effective mobility of μ_effective_ = 86.0 cm^2^ V^−1^ s^−1^. This dimensionless factor, defined as the ratio of the maximum channel conductivity of an equivalent ideal transistor to that experimentally achieved in the device, provides a stringent criterion for assessing the robustness of mobility extraction. Detailed procedures for calculating *r* and determining the effective mobility (μ_effective_ = *r* × μ_FE_) are provided in the note S1 (*40*). The estimated *r* values for all three cases are close to 1. The corresponding μ_effective_ values, together with *r*, μ_FE_, and *I*_on/off_ values for all three cases, are summarized in table S3.

Subthreshold swing (SS) values follow a similar trend (fig. S14 and table S3). Compared with pristine devices, which show an SS of 0.76 ± 0.02 V dec^−1^, NH_4_SCN-treated devices exhibit a reduced SS of 0.64 ± 0.12 V dec^−1^, while GuaSCN-treated devices achieve the lowest SS of 0.51 ± 0.08 V dec^−1^, approaching more ideal switching behavior. This progressive reduction in SS correlates strongly with the improved film morphology and diminished trap densities achieved through SCN-additive-mediated enhancement of the perovskite crystal structure.

The improved crystallinity of the GuaSCN-modified perovskite also translates into superior operational stability. In p-channel Sn halide perovskite TFTs, it is known that electron traps (e.g., Frenkel-type defects such as iodide vacancy/interstitial pairs), filled during the reverse (on-to-off) scan, induce anticlockwise hysteresis: trapped negative charges shift the *V*_T_ of the subsequent forward-scan transfer curve toward more positive values (*30*). Incorporation of GuaSCN additive markedly mitigates this hysteresis between forward and reverse sweeps (Fig. 3, B and C and fig. S11A). To further evaluate ion-migration-related hysteresis behavior, scan-rate-dependent dual-scan transfer measurements were additionally performed for the optimized GuaSCN-treated TFTs (fig. S15A). The transfer characteristics remain nearly unchanged across different gate-voltage sweep rates, with only minimal separation between the forward and reverse scans. The hysteresis voltage (Δ*V*_Hysteresis_), extracted from 30 devices at each scan rate, also shows weak scan-rate dependence over the investigated range (fig. S15B), indicating suppressed ionic redistribution dynamics in the optimized perovskite channel. In addition, neither the field-effect mobility nor the maximum drain current exhibits a systematic dependence on the gate-voltage sweep rate over the investigated range (fig. S15, C and D). These results are consistent with the enhanced iodide migration barrier predicted by DFT calculations.

The GuaSCN-incorporated devices exhibit highly stable behavior, with nearly negligible changes in *V*_T_ throughout repeated sweep sequences. During continuous 500-cycle forward–reverse sweep measurements (Fig. 3H), the transfer characteristics remain essentially unchanged, with minimal variation in on/off current or subthreshold slope. The absence of hysteresis growth or current degradation indicates that trapping centers are strongly suppressed and that the electronic landscape of the perovskite channel remains stable under sustained gate-field modulation (*38*). We additionally monitored the threshold-voltage evolution during the repeated switching cycles (500 cycles) (fig. S16). The pristine TFTs exhibit progressively increasing threshold-voltage drift during cycling, whereas the GuaSCN-treated TFTs maintain substantially more stable threshold voltages with significantly suppressed drift behavior. These results further support that GuaSCN suppresses defect- and ion-migration-induced operational instability within the Sn halide perovskite channel.

Bias-stress testing further supports this stability. Under a constant gate bias of 40 V for up to 36,000 s, the GuaSCN-treated TFTs maintain nearly unchanged dual-scan transfer characteristics, including both forward and reverse gate-voltage sweeps, with minimal hysteresis evolution (Fig. 3I). The hysteresis voltage (Δ*V*_Hysteresis_) was extracted as a function of stressing time (fig. S17), and only a slight increase in Δ*V*_Hysteresis_ is observed during prolonged gate-bias operation, followed by gradual stabilization. This long-term invariance highlights the suppression of mobile ionic species and the reduced reconfiguration of deep defect states, which are typically responsible for bias-induced degradation in Sn-based perovskite transistors (*41*). Long-term on/off switching endurance (Fig. 3J) provides additional support: under alternating gate pulses (*V*_GS_ = −40 V for on and + 40 V for off; pulse width = 1 s; *V*_DS_ = −40 V) for 12,000 cycles, both on- and off-state currents remain highly stable with no observable drift, underscoring the suppression of charge trapping/detrapping processes even under rapid, high-amplitude gate stress.

Meanwhile, potential non-equilibrium effects associated with the high Joule power and current densities in our TFTs were evaluated. In addition to examining the absence of systematic sweep-rate dependence (fig. S15) and the highly reversible electrical characteristics during repeated operation (Fig. 3, H and I), we measured the output characteristics using drain-voltage pulses with different pulse widths and compared them with those obtained from conventional dc sweeps (Fig. 3F and fig. S18). Even when the electrical excitation duration was reduced by a factor of two, the drain current varied by no more than 1.8%, indicating that self-heating does not appreciably affect the measured output current within the investigated bias and pulse-width ranges. Collectively, these results indicate that the reported field-effect mobility and on-state current are robust against measurement-timescale-dependent non-equilibrium effects under the investigated conditions. Further details are provided in note S2.

To further assess the uniformity of the optimized condition, Fig. 3K shows forward-scan transfer curves from 300 GuaSCN-treated devices, along with the corresponding histogram of extracted *V*_T_ values (fig. S19). The highly overlapping traces demonstrate excellent device-to-device reproducibility and confirm that GuaSCN-assisted crystallization yields perovskite films with uniform electronic properties suitable for scalable device arrays.

Beyond operational stability, we evaluated the long-term shelf stability of the optimized GuaSCN-treated TFTs by monitoring their transfer characteristics over 100 days in ambient air under glass encapsulation (Fig. 3L). The encapsulated devices exhibit only minimal variations in *V*_T_, subthreshold slope, and on-state current over the entire storage period, indicating that the GuaSCN-engineered perovskite lattice remains structurally and electronically robust over extended timescales. To further distinguish the intrinsic stabilization effect of GuaSCN from the environmental protection effect of glass encapsulation, additional accelerated aging measurements were conducted under encapsulated conditions following the methodology used in our previous MACl-substituted Sn perovskite TFT study (*30*). The degradation kinetics were evaluated at elevated temperatures (80°, 100°, and 120°C), and the characteristic lifetime was extracted using a 30% retention criterion (fig. S20). Arrhenius analysis yielded an extrapolated room-temperature operational lifetime of approximately 16.1 years for the encapsulated GuaSCN-treated TFTs, indicating substantially suppressed degradation kinetics, relative to the pristine device (10.4 years) (*30*). Furthermore, non-encapsulated devices exposed to ambient air for 5 min also exhibited markedly different behaviors (fig. S21). While pristine TFTs underwent severe current degradation after air exposure, the GuaSCN-treated devices retained stable transfer characteristics with substantially suppressed degradation. Taken together, these results demonstrate that SCN engineering—especially with GuaSCN—provides comprehensive stability benefits, delivering perovskite TFTs with both superior operational endurance and long-term reliability.

## DISCUSSION

We have established a design principle for non-metallic SCN-based additives that simultaneously enhances the structural robustness, optoelectronic quality, and device–level performance of Sn halide perovskites. Using DFT calculations, we demonstrate that the local environment of FASnI_3_ is highly sensitive to the identity of the A–site cation when iodide is replaced by SCN^**−**^. As a model additive, we select GuaSCN, which reinforces the inorganic framework by enabling stronger Sn–X–Sn bonding—validated both theoretically and experimentally—while its strong hydrogen bonding between the A-site cation and iodine impedes I^−^ migration, a major origin of instability in Sn halide perovskites. Furthermore, incorporation of GuaSCN into FASnI_3_ leads to pronounced improvements in crystallinity, enlarged grains, and a substantial reduction in nonradiative recombination, accompanied by clear suppression of Sn^2+^ oxidation. These improvements are achieved without significant changes in the bandgap or overall electronic structure.

When implemented as active channels in bottom-gate, top-contact TFTs, GuaSCN-treated FASnI_3_ films deliver a step change in p-channel performance. The optimized devices exhibit μ_FE_ above 88 cm^2^ V^**−**1^ s^**−**1^, *I*_on/off_ exceeding 4 × 10^9^, and subthreshold swings as low as 0.51 V dec^−1^, with negligible hysteresis between forward and reverse sweeps. These metrics, combined with excellent device-to-device reproducibility across 300 devices, demonstrate that SCN-mediated crystallization can yield uniform, high-quality perovskite semiconductors in ultrathin channels. Moreover, the devices show remarkable operational robustness under repeated gate sweeps, prolonged negative-bias stress, and long-term on/off pulsing, as well as outstanding shelf stability over 100 days under ambient storage with encapsulation. The absence of performance degradation under these demanding conditions points to effective suppression of trap formation, ion migration, and defect reconfiguration in the GuaSCN-engineered perovskite lattice.

Overall, our study reveals that SCN^**−**^ in Sn halide perovskites should be regarded not merely as a generic pseudohalide additive, but as a multidimensional design parameter that must be considered in concert with the co-introduced A-site cation. The A–site–pseudohalide co–design concept introduced here provides a general strategy for tailoring both the electronic and ionic landscapes of Sn-based perovskites. By bridging atomistic design rules with record-level p-channel transistor performance and stability, this work advances Sn halide perovskites toward practical, lead-free, high-mobility electronics.

## MATERIALS AND METHODS

### Materials

Formamidinium iodide (FAI, >95%) was obtained from GreatCell Solar Materials. Methylammonium chloride (MACl) was sourced from Xi’an Polymer Light Technology Corp. Tin (II) fluoride (SnF_2_, 99%), metallic tin powder (99.5%), anhydrous *N,N*-dimethylformamide (DMF, 99.8%), anhydrous dimethyl sulfoxide (DMSO, 99.5%), anhydrous chlorobenzene (CB, 99.8%), ammonium thiocyanate (NH_4_SCN, ≥99.99%), and guanidinium thiocyanate (GuaSCN, ≥99.99%) were all sourced from Sigma-Aldrich. Ultra-dry tin(II) iodide (SnI_2_, 10-mesh beads, 99.999%) was supplied by Alfa Aesar. Gold pellets (Au, 99.99%) were obtained from RND Korea. All reagents were used as received without further purification. All precursor handling was conducted in a nitrogen-filled glovebox unless otherwise noted.

### Preparation of the precursor solution

FA_0.85_MA_0.15_SnI_2.85_Cl_0.15_ perovskite precursor solution was prepared by dissolving FAI (0.17 M), MACl (0.03 M), and SnI_2_ (0.2 M) in a mixed solvent of DMF and DMSO (4:1 v/v) to obtain a final concentration of 0.2 M. To suppress unintentional oxidation of Sn^2+^, 10 mol% SnF_2_ (relative to SnI_2_) and metallic Sn powder (5 mg ml^−1^) were added to the precursor solution (*42*). Thiocyanate-based additives were incorporated by introducing NH_4_SCN or GuaSCN at 2.5 mol% with respect to SnI_2_. All precursor mixtures were stirred at room temperature for 2 h in a nitrogen-filled glovebox (O_2_/H_2_O < 1 ppm). Before film deposition, the solution was passed through a 0.2 μm PTFE syringe filter to remove any undissolved particulates. All precursor solutions were used immediately after filtration to minimize moisture uptake and prevent oxidation-related degradation.

### Device fabrication and characterization

Bottom-gate, top-contact TFTs were fabricated on highly doped p^+^-Si substrates with a 300-nm thermally grown SiO_2_ dielectric layer, respectively. Prior to film deposition, the substrates were cleaned sequentially in acetone, isopropanol, and deionized water, followed by N_2_ drying and 15 min of UV–ozone treatment to promote surface cleanliness and wettability. Perovskite layers were deposited inside a nitrogen-filled glovebox. The precursor solution (80 μl) was dispensed onto the substrates and spin-coated using a one-step program of 8,000 rpm for 60 s. Next, 100 ul of chlorobenzene was dripped onto the spinning substrate 15 s after the spin began. After deposition, the films were thermally annealed at 80°C for 10 min and subsequently cooled to room temperature under inert conditions. Gold electrodes were deposited by thermal evaporation through a shadow mask under a base pressure below 3 × 10^−6^ Torr. The electrode thickness was set to 50 nm, defining a channel length of 100 μm and a channel width of 1500 μm. All electrical characterizations of the TFT devices were performed in ambient air and in the dark after glass encapsulation, except for the measurement shown in fig. S21, which were intentionally performed without glass-encapsulation. Optical photographs of the glass-encapsulated TFT devices and enlarged device region are provided in fig. S22. The right panel of fig. S22 shows tip-scratches introduced prior to measurement. The slight upward current observed at high positive gate voltages in the transfer curves originated from the long metal contact lines used for encapsulation and disappeared upon cutting those lines (*30*). The TFT devices were encapsulated inside a nitrogen-filled glovebox using a glass coverslip sealed with UV-curable epoxy (NOA-68). The encapsulation fully covered the active TFT region, while the external Au probing pads were intentionally left exposed to allow electrical characterization without removing the encapsulation.

### Perovskite film and device characterizations

X-ray diffraction (XRD) measurements were carried out on an X-ray diffractometer (D8 Advance, Bruker) equipped with a Cu Kα irradiation (λ = 1.5406 Å). Diffraction patterns were collected in the 2θ range of 10°–40° with a step size of 0.03° and a scan rate of 1.5° min^−1^. For these measurements, perovskite layers were deposited on Si/SiO_2_ substrates following the same procedures used for device fabrication. Scanning electron microscopy (FE-SEM, S-4800, Hitachi) was used to evaluate film coverage with images acquired at accelerating voltage of 10 kV. X-ray photoelectron spectroscopy (XPS) was performed using a K-Alpha plus system (Thermo Scientific) with Al Kα X-ray (12 keV/6 mA, 400 μm spot size). To minimize uncertainties arising from surface oxidation of the Sn-based perovskite layer, samples were etched for 25 s using a 1 keV Ar^+^ cluster ion beam. Fourier-transform infrared (FT-IR) spectra were acquired using a Nicolet 6700 spectrometer (Thermo Scientific) operated in attenuated total reflectance (ATR) mode. Optical absorption spectra were recorded in the range of 600–1000 nm using a UV-2600 spectrophotometer (SCINCO). Steady-state photoluminescence (PL) spectra were collected with a QM-400 spectrofluorometer (HORIBA) equipped with an R928P photomultiplier detector. A 635 nm excitation source was used, and emission spectra were acquired from 700–1000 nm using an 8 nm slit. Time-resolved PL (TRPL) traces were measured on a time-correlated single-photon counting system (Fluorolog-QM, HORIBA) with a 635 nm picosecond-pulsed diode laser (Xe arc lamp, 75 W). Decay dynamics were monitored at the emission maximum (878 nm for the corresponding compositions). Time-of-flight secondary ion mass spectrometry (ToF-SIMS) depth profiling was carried out using a ToF-SIMS 5 instrument (ION-TOF GmbH, Germany) to investigate the depth distribution of additive-derived species within the perovskite films. A Bi^+^ primary ion beam (30 keV and 1 pA) was employed for secondary-ion detection, while sputtering was performed using a Cs^+^ ion beam operated at 0.5 keV and 40 nA. Depth profiles were obtained through alternating sputtering and acquisition cycles consisting of 1 s sputtering followed by 1.7 s signal collection. The sputtering and analysis areas were set to 500 by 500 μm^2^ and 100 by 100 μm^2^, respectively, to minimize crater-edge effects during depth profiling. Unless otherwise noted, all electrical measurements were performed in ambient air (relative humidity 35–45%) on glass-encapsulated TFTs. Devices were characterized using a semiconductor parameter analyzer (Keithley 2636B) connected to a probe station inside a dark-shielded enclosure to eliminate light-induced artifacts. To evaluate possible self-heating effects under high-current operation, pulsed-*I–V* measurements were performed with different drain-voltage pulse widths. During the pulsed measurements, the gate voltage was held constant at *V*_GS_ = −40 V, while voltage pulses were applied to the drain. Drain-voltage pulse widths of 1000, 750, and 500 μs were used, and the resulting output characteristics were compared with those obtained from conventional dc-sweep measurements under the same gate-bias condition. Reducing the pulse width shortens the duration of electrical excitation and thereby limits the time available for thermal accumulation during the on state.

### Computational Details

DFT calculations were performed using QM/MM simulation cluster [NFEC-2025-02-303332] and the Vienna ab initio Simulation Package (VASP) (*43*). Exchange-correlation energies were treated by Perdew-Burke-Ernzerhof (PBE) functional based on generalized gradient approximation (GGA) (*44*). The simulation models were constructed with a pseudocubic structure (*a* = *b* = *c* = 6.29 Å, α = β = γ = 90.00°) for FASnI_3_. Although our experiments used FA_0.85_MA_0.15_SnI_2.85_Cl_0.15_, it is still challenging to consider all possible configurations within the supercell at the atomic scale due to the limitations of computational cost. In addition, we also confirmed that FA sites are more favorable (lower substitutional energy for FA relative to MA) for substitution by other cations in FA_0.625_MA_0.375_SnI_2.875_Cl_0.125_ (fig. S23). Thus, we used FASnI_3_-based models to focus on the effects of A-site substitution and SCN incorporation. Substitutional energy (*E*_sub_, fig. S23) of FA or MA with an additive A-site cation was calculated by *E*_sub_ = (*E*_total_ + *E*_cat_0_) – (*E*_0_ + *E*_cat_), where *E*_total_ is the total energy of the A-site substituted FA_0.625_MA_0.375_SnI_2.875_Cl_0.125_ system, *E*_cat_0_ is the total energy of MA or FA cations in vacuum, *E*_0_ is the total energy of pristine FA_0.625_MA_0.375_SnI_2.875_Cl_0.125_, and *E*_cat_ is the total energy of NH_4_ or Gua cations in vacuum. The lower *E*_sub_ values for FA relative to MA indicate that FA sites are more favorable for substitution by other cations. A 2 by 2 by 2 supercell was used to represent the FA_0.875_A_0.125_SnI_3_ or FA_0.875_A_0.125_SnI_2.875_SCN_0.125_, in which A represents the different A-site cations. To further validate the experimental composition containing MACl, additional calculations were performed using FA_0.875_MA_0.125_SnI_2.85_Cl_0.15_, the closest approximation to the experimental composition (FA_0.85_MA_0.15_SnI_2.85_Cl_0.15_). A 3 by 3 by 3 Monkhorst–Pack k-point mesh was used for Brillouin zone sampling (*45*). A plane wave was expanded with a cutoff of 400 eV and convergence criteria of 10^−4^ eV for relaxation and 10^−6^ eV for electronic structure. Gaussian smearing was used with a width of 0.05 eV to determine partial occupancies. Geometries were relaxed using a conjugate gradient (CG) algorithm until the forces were less than 0.03 eV/Å. DFT-D3 was used to correct for van der Waals interactions (*46*, *47*). The crystal orbital Hamilton population (COHP) was calculated using LOBSTER software to analyze the chemical bond characteristics (*48*). The migration barrier of iodine was calculated by using the climbing image-nudge elastic band (CI-NEB) method with three intermediate images to determine the minimum energy pathway (MEP) (*49*). The volume of the cuboctahedral cavity defined by the 12 anions surrounding the A-site cation was calculated using a convex hull algorithm (SciPy) (*50*).

## Supplementary Materials

### This PDF file includes:

Figs. S1 to S23 Supplementary Notes 1 and 2 Tables S1 to S3 References
