# Supplementary material for: A-site cation–pseudohalide co-design for tin halide perovskite transistors
Source: Sci Adv. 2026 Oct 30;12(44):eaeh2062. doi: 10.1126/sciadv.aeh2062 (PMC13721964; doi:10.1126/sciadv.aeh2062)
Supplement: Supplementary file 1 [file sciadv.aeh2062_sm.pdf]

Science Advances
AAAS

Supplementary Materials for

A-site cation–pseudohalide co-design for tin halide perovskite transistors

Bum Ho Jeong *et al.*

Corresponding author: Kyeounghak Kim, chemekim@hanyang.ac.kr; Hui Joon Park, huijoon@hanyang.ac.kr

Sci. Adv. **12**, eaeh2062 (2026)
DOI: 10.1126/sciadv.aeh2062

This PDF file includes:

Figs. S1 to S23
Supplementary Notes 1 and 2
Tables S1 to S3
References

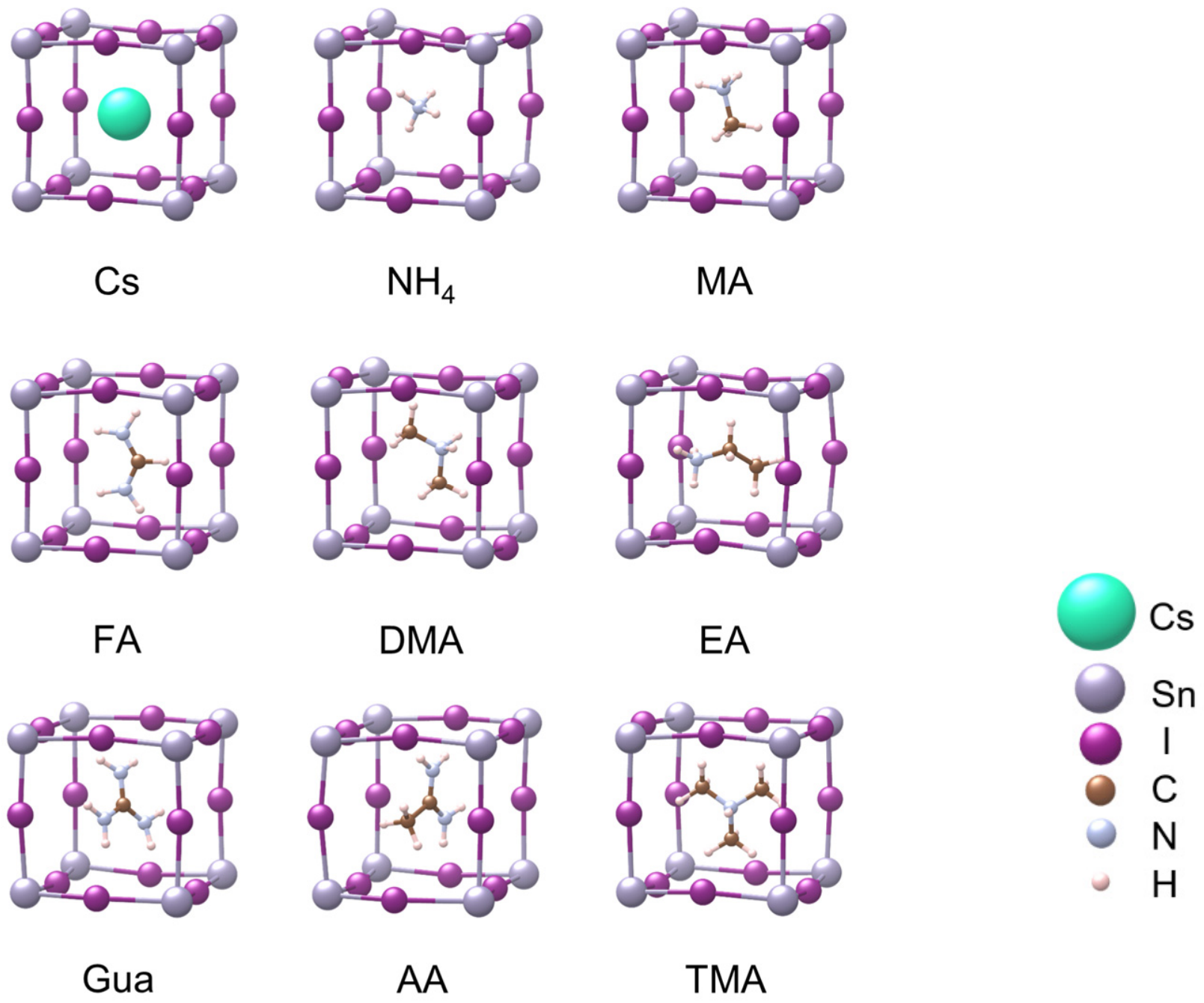

Fig. S1.

Local environments of the optimized $FASnI_3$ with the addition of different A-site cations in the absence of SCN. The abbreviations used for A-site cations are as follows: Cs (cesium), NH_4 (ammonium), MA (methylammonium), FA (formamidinium), DMA (dimethylammonium), EA (ethylammonium), Gua (guanidinium), AA (acetamidinium), and TMA (trimethylammonium).

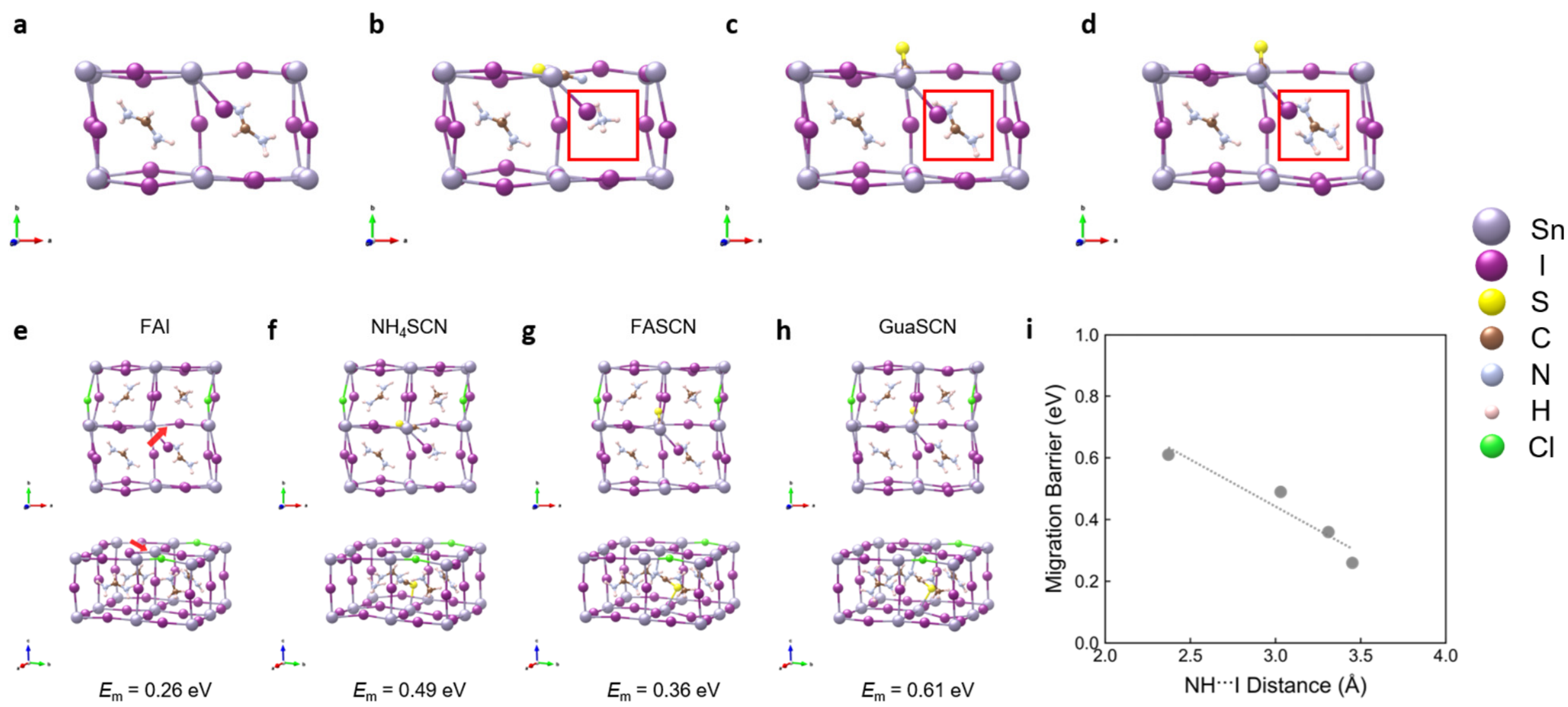

Fig. S2.

Hydrogen-bonding environments and iodide migration barriers. Local coordination environments surrounding the migrating iodide ion in $FA_{0.875}MA_{0.125}SnI_{2.85}Cl_{0.15}$ with (**A**) FAI, (**B**) NH_4SCN, (**C**) FASCN, and (**D**) GuaSCN. The red boxes indicate the neighbouring A-site cations that interact with the migrating iodide ion along the migration pathway. Transition-state structures for iodide migration in $FA_{0.875}MA_{0.125}SnI_{2.85}Cl_{0.15}$ with (**E**) FAI, (**F**) NH_4SCN, (**G**) FASCN, and (**H**) GuaSCN. The red arrows in (**E**) indicate the migration direction. The migration barriers (E_m) are indicated below each structure. (**I**) Correlation between the hydrogen bond distance at transition state and migration barrier.

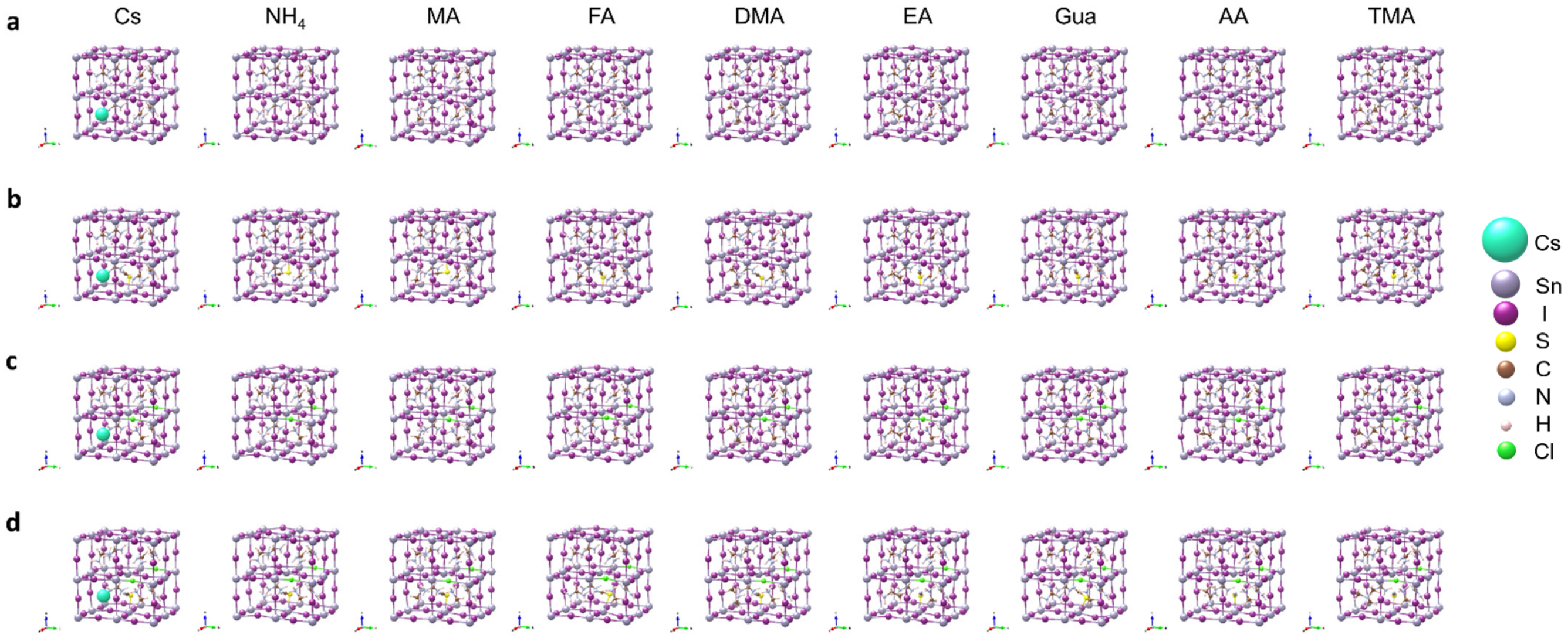

Fig. S3.

Optimized structures with different A-site cations and SCN additives. Optimized structures of $FASnI_3$ with various A-site cations (**A**) without and (**B**) with SCN additive, and those of $FA_{0.875}MA_{0.125}SnI_{2.85}Cl_{0.15}$ with various A-site cations (**C**) without and (**D**) with SCN additive. For reference, due to the finite size of the DFT supercell, the composition was approximated as $FA_{0.875}MA_{0.125}SnI_{2.85}Cl_{0.15}$, which is the closest representation to the experimental composition ($FA_{0.85}MA_{0.15}SnI_{2.85}Cl_{0.15}$).

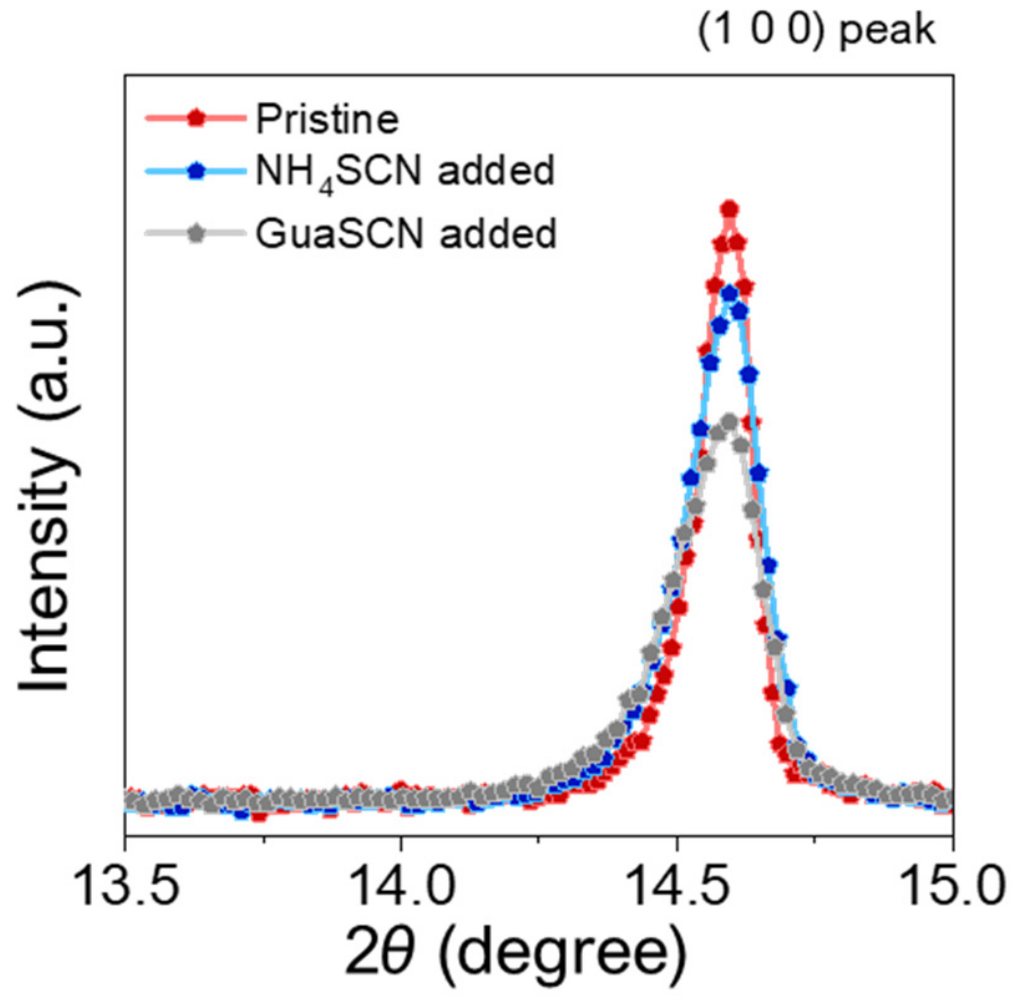

Fig. S4.

Enlarged XRD patterns of the (100) reflection for pristine $FASnI_3$, NH_4SCN-treated $FASnI_3$, and GuaSCN-treated $FASnI_3$ films. The (100) reflection shows progressive peak narrowing without a meaningful peak-position shift, consistent with the trend observed for the (200) reflection.

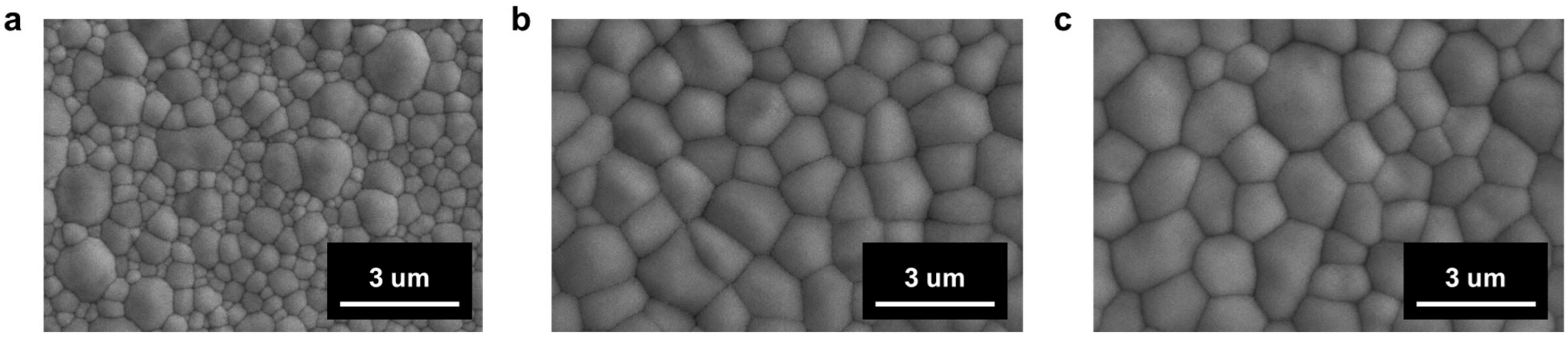

Fig. S5.

Low-magnification SEM image of SCN-based additive-treated $FASnI_3$ films. (**A**) $FASnI_3$, (**B**) NH_4SCN-treated $FASnI_3$, and (**C**) GuaSCN-treated $FASnI_3$ films.

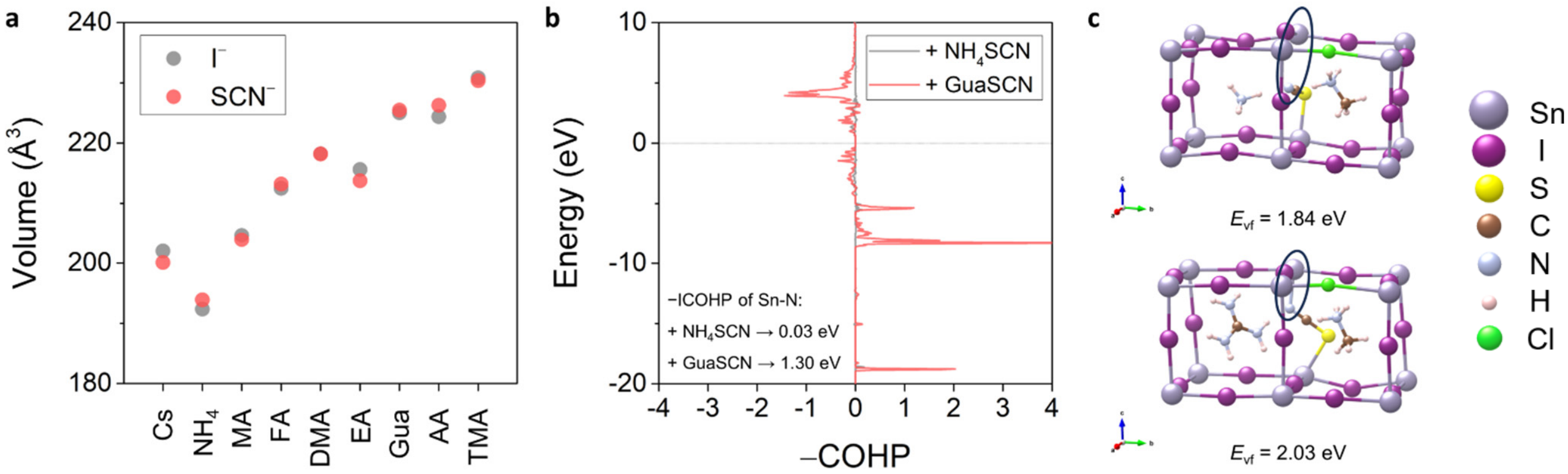

Fig. S6.

Relationship between local structure, Sn–N bonding, and Sn-vacancy formation. (**A**) Volume of the cuboctahedral cavities for different A-site cations containing I^- (grey) and SCN^- (red) in $FA_{0.875}MA_{0.125}SnI_{2.85}Cl_{0.15}$. (**B**) −COHP of the Sn–N interaction in $FA_{0.875}MA_{0.125}SnI_{2.85}Cl_{0.15}$ with NH_4SCN and GuaSCN. (**C**) Representative local structures of the NH_4SCN- and GuaSCN-treated systems. The black circled regions highlight the Sn–N coordination environment, with the corresponding Sn-vacancy formation energies (E_{vf}).

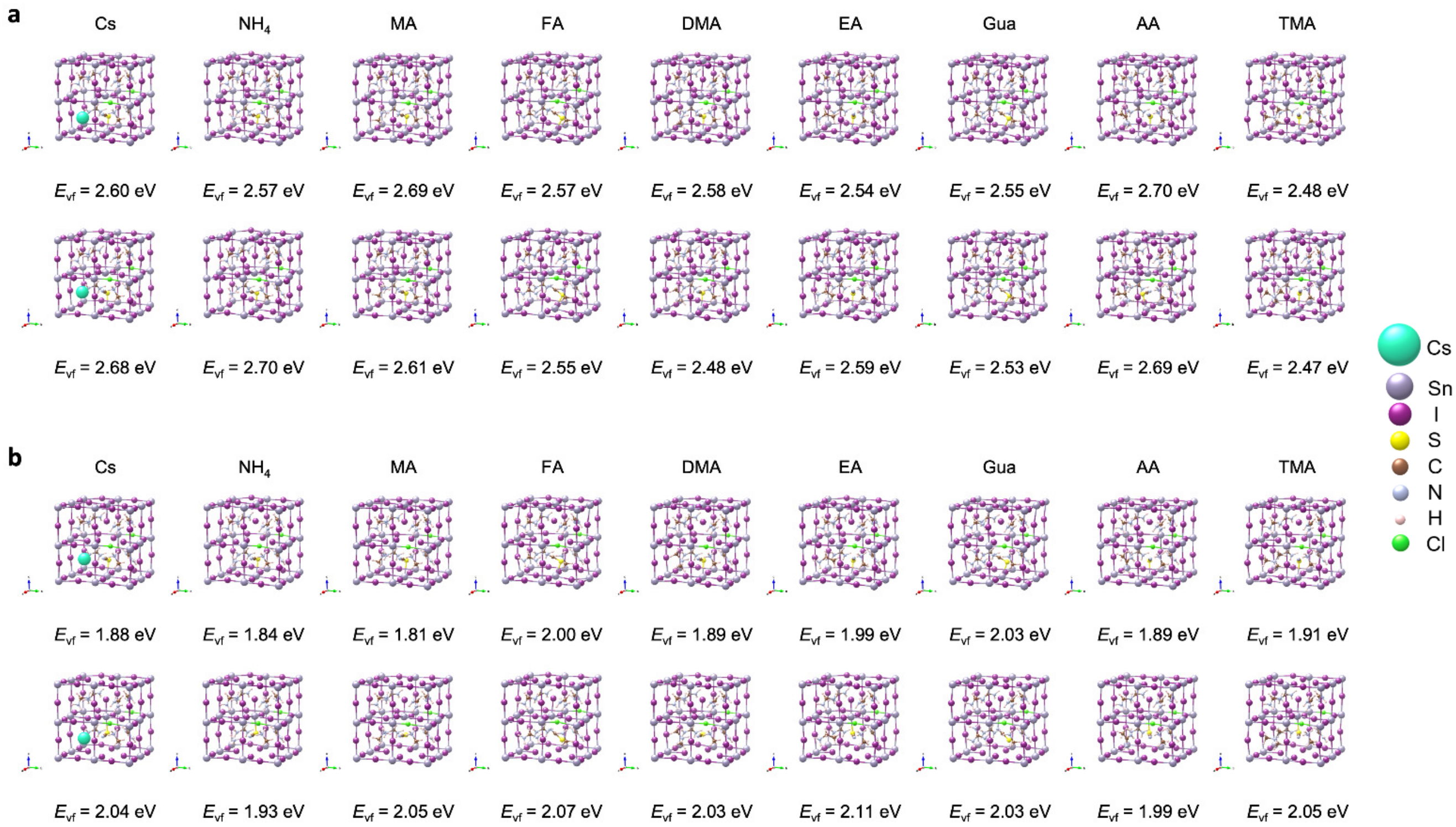

Fig. S7.

Iodine and tin vacancy formation energies with different A-site cations. Optimized structures and vacancy formation energies (E_{vf}) of (**A**) two different iodide-vacancy sites and (**B**) two different Sn-vacancy sites in $FA_{0.875}MA_{0.125}SnI_{2.85}Cl_{0.15}$ with different A-site cations. For the Sn vacancies in (**B**), the upper and lower panels correspond to vacancies formed at the Sn sites coordinated to the N and S ends of SCN, respectively.

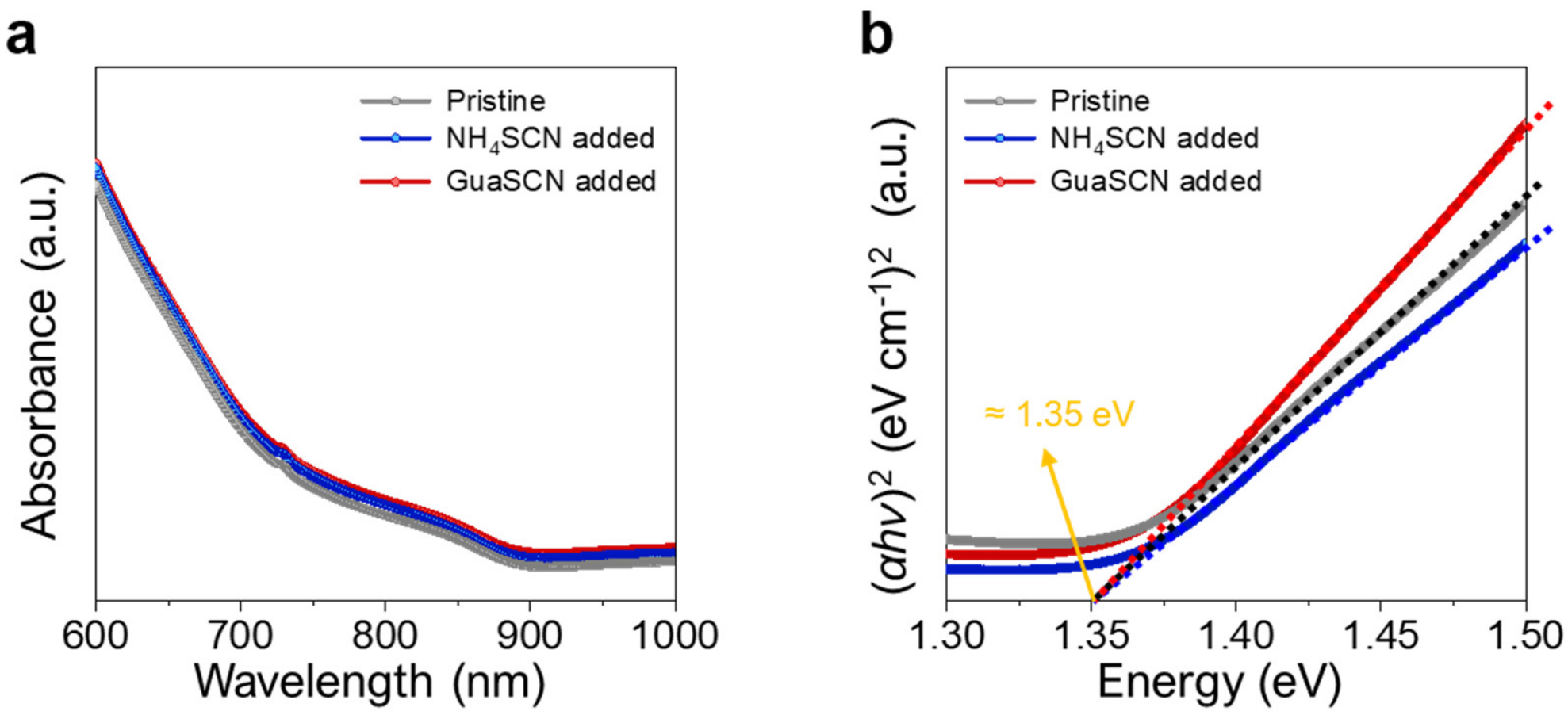

Fig. S8.

Optical absorption and bandgap analysis of SCN-treated $FASnI_3$ films. (**A**) UV-vis absorption spectra of $FASnI_3$, NH_4SCN-treated, and GuaSCN-treated $FASnI_3$ films. (**B**) Tauc plots extracted from the UV-vis spectra, with fitting lines showing that the optical bandgap remains essentially unchanged across all compositions.

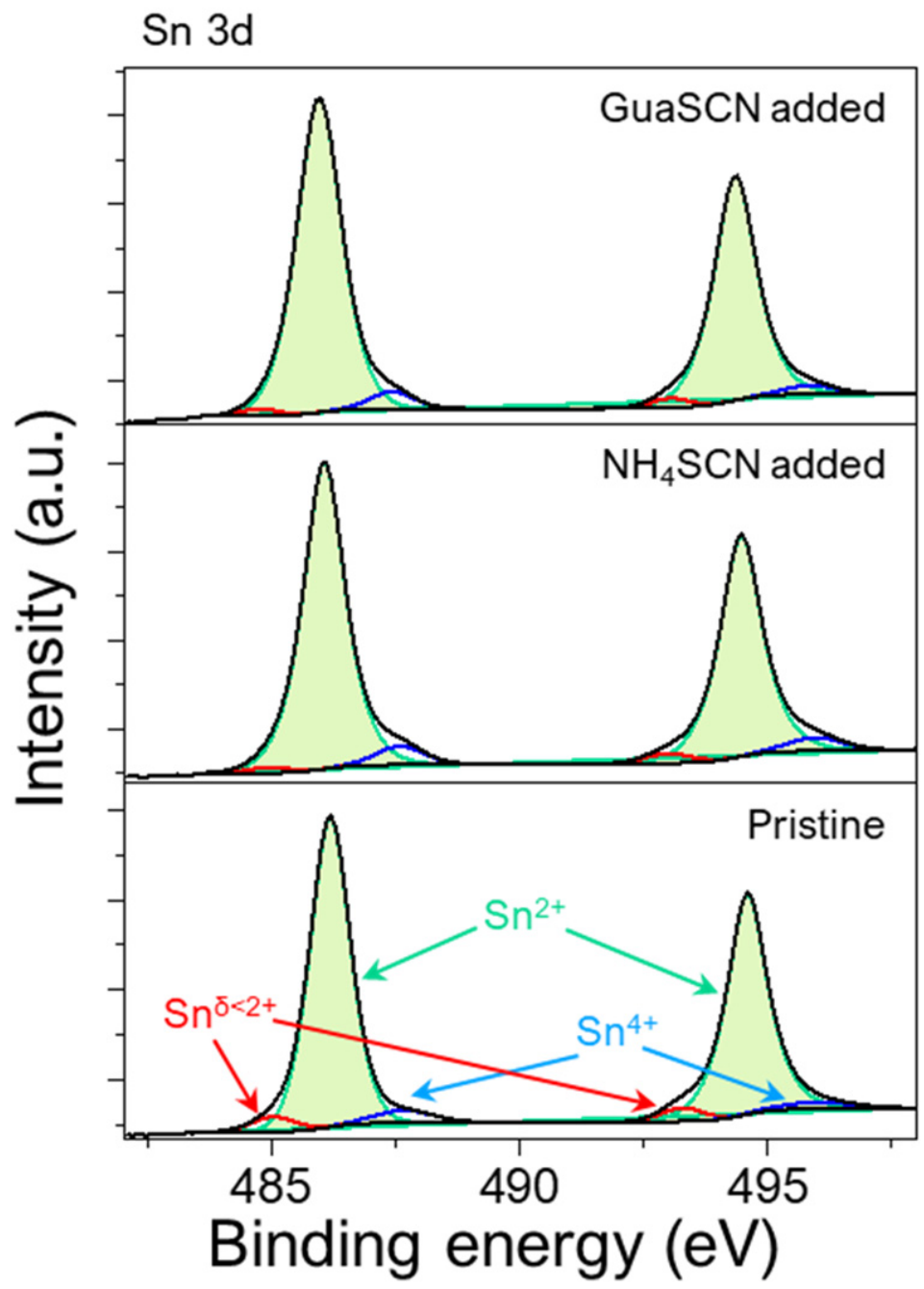

Fig. S9.

Sn oxidation-state analysis by high-resolution XPS. High-resolution Sn 3d XPS spectra with peak deconvolution for $FASnI_3$, NH_4SCN-treated, and GuaSCN-treated $FASnI_3$ films.

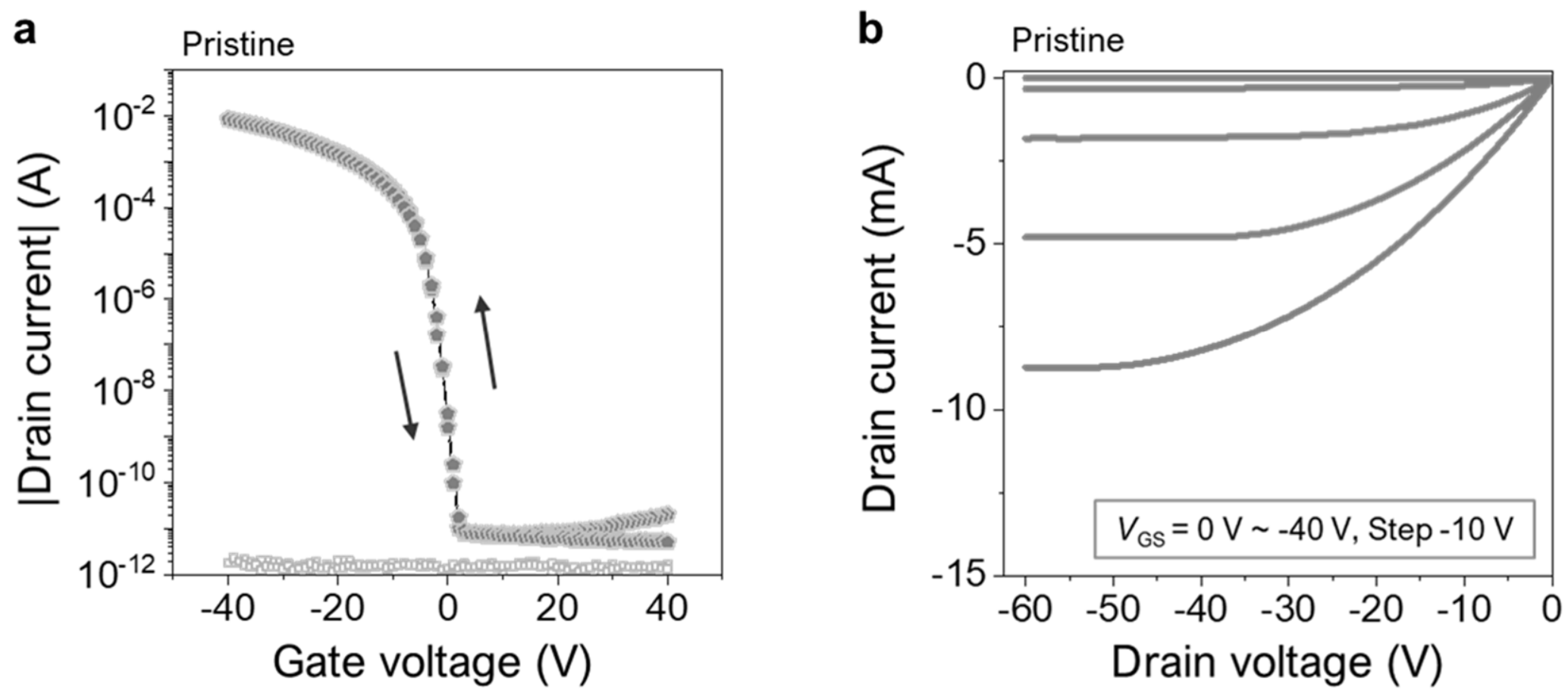

Fig. S10.

Electrical characteristics of $FASnI_3$ thin-film transistors. (**A**) Dual-scan transfer characteristics of $FASnI_3$ TFT without SCN-based additive measured at $V_{DS} = -40$ V, exhibiting an average μ_{FE} of 73.01 ± 8.03 $cm^2\ V^{-1}\ s^{-1}$ and an average $I_{on/off}$ of $(1.02 \pm 0.16) \times 10^9$. The corresponding gate current (I_G) characteristics are also plotted on a logarithmic scale. (**B**) Corresponding output characteristics.

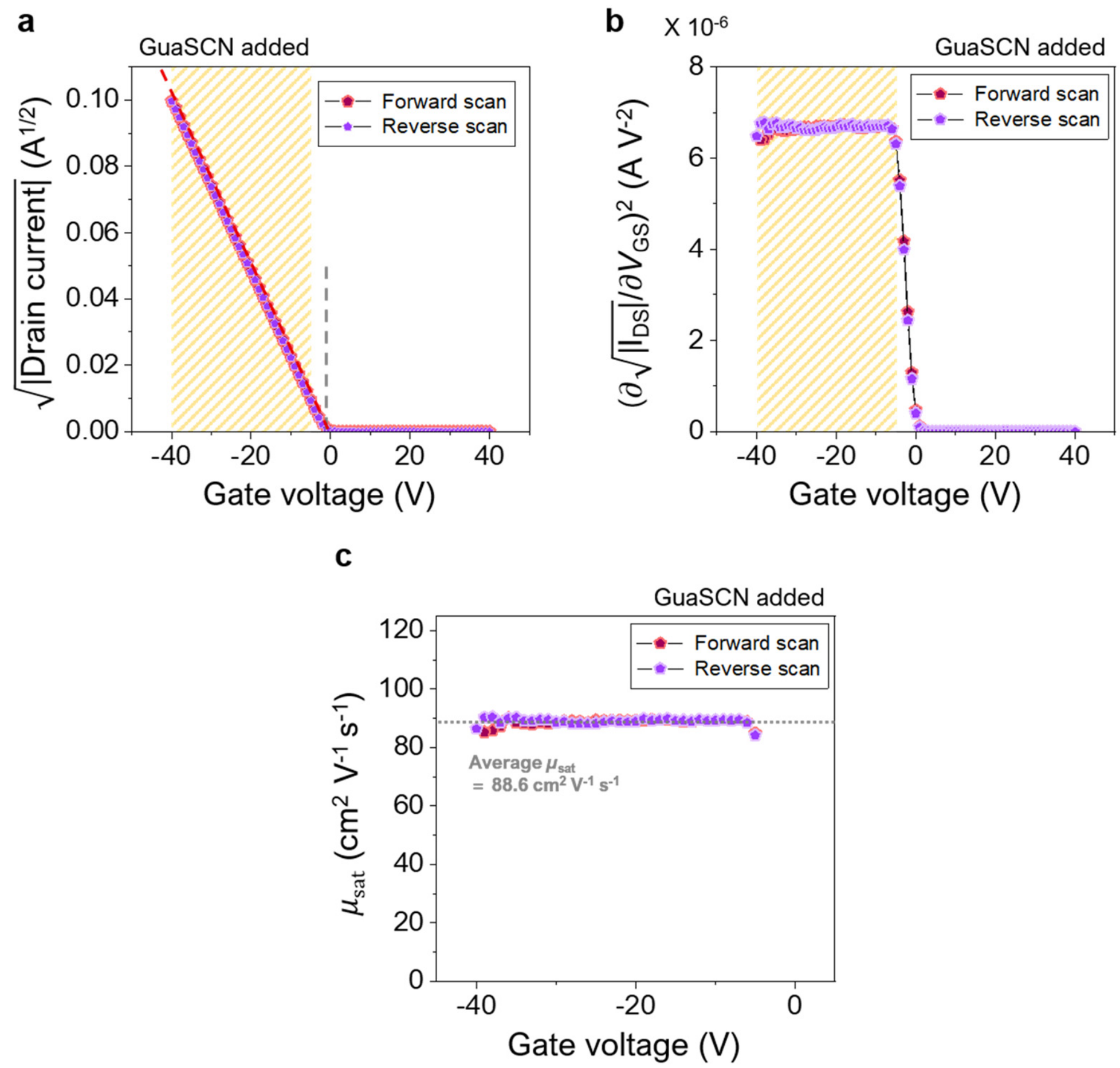

Fig. S11.

Gate-voltage-dependent field-effect mobility analysis and validation. (**A**) Double-linear plot of $\sqrt{|I_{DS}|}$ versus V_{GS} for the best-performing GuaSCN-treated TFT measured under forward and reverse scans at V_{DS} = -40 V (same device as in Fig. 3B). The hatched region indicates the gate-voltage range used for mobility extraction (-40 ≤ V_{GS} ≤ -5 V), and the red dashed line represents the linear fit to the forward-scan data within this range. The gray dashed line marks the upper boundary of the fitting range at V_{GS} = -5 V. (**B**) Corresponding $\left(\partial\sqrt{|I_{DS}|}/\partial V_{GS}\right)^2$ values extracted from the transfer characteristics in (A). (**C**) Variation of the field-effect mobility (μ_{sat}) as a function of V_{GS} in the saturation regime, calculated from the data in (B).

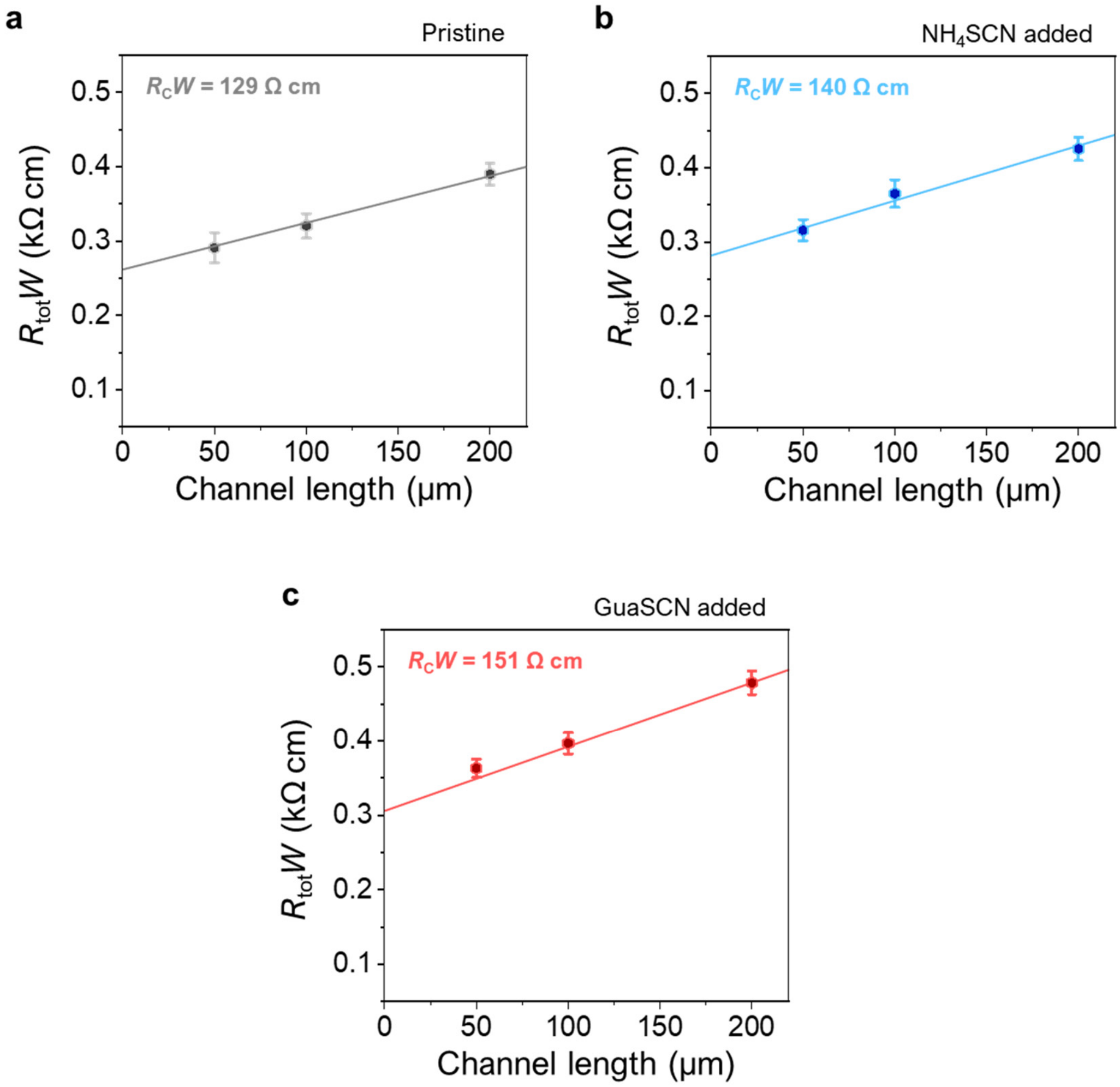

Fig. S12.

Contact resistance analysis. Transmission-line method for contact resistance calculations for (**A**) pristine, (**B**) NH_4SCN-treated, and (**C**) GuaSCN-treated TFTs. Contact resistance (R_cW) values are estimated by the y-axis intercepts and the drain currents collected from the *I-V* curve (i.e., output curve) over the range from 0 V to -5 V at V_{GS} = -40 V. Symbols and error bars indicate the mean and standard deviation, calculated from 100 devices under each condition.

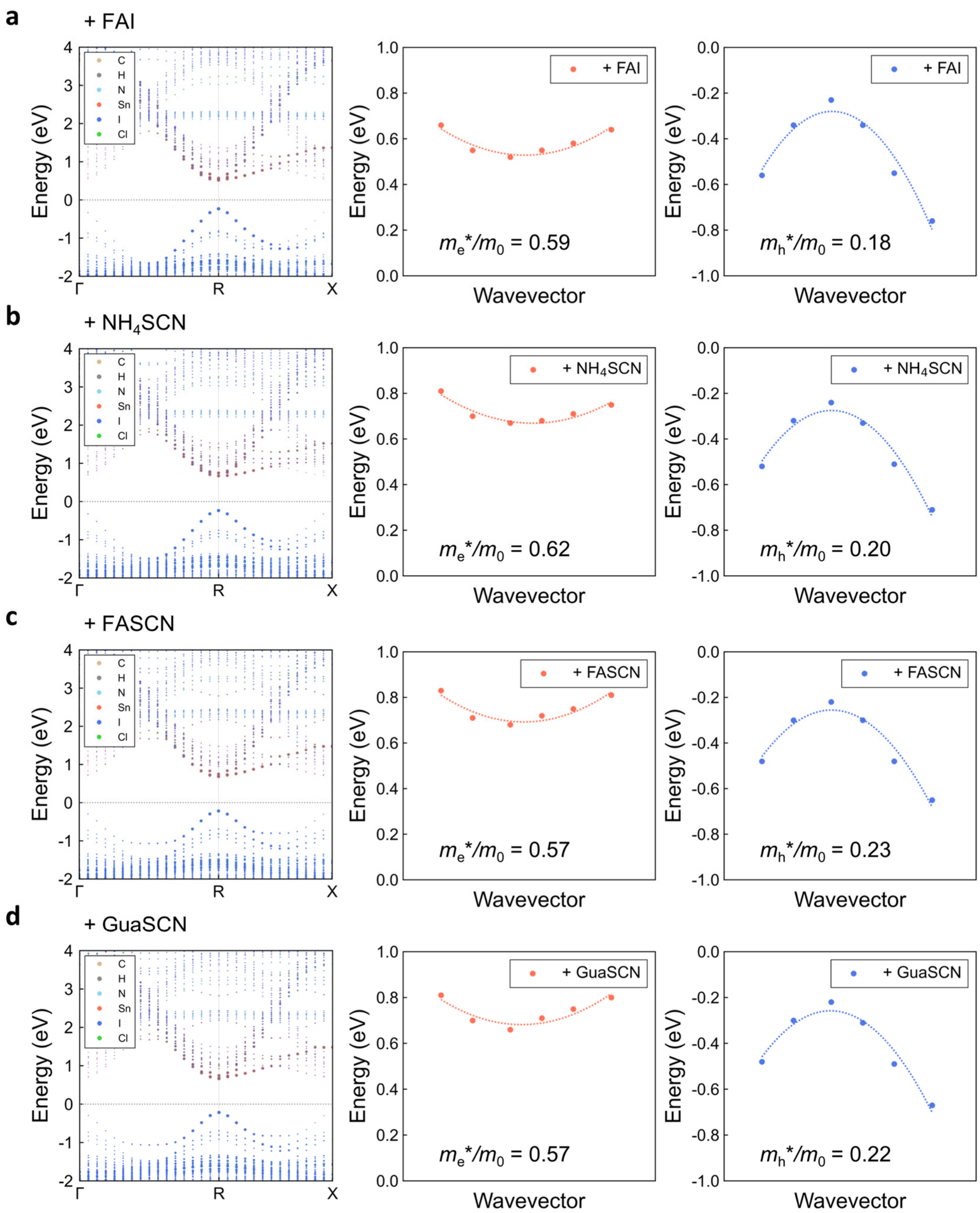

Fig. S13.

Electronic band structures and effective-mass calculations. Electronic band structures of $FA_{0.875}MA_{0.125}SnI_{2.85}Cl_{0.15}$ with (**A**) FAI, (**B**) NH_4SCN, (**C**) FASCN, and (**D**) GuaSCN. Quadratic fitting of the conduction band minimum (CBM) and valence band maximum (VBM) near the band edges is shown for the calculation of the electron and hole effective masses (m_e^* and m_h^*). m_0 denotes the free-electron mass.

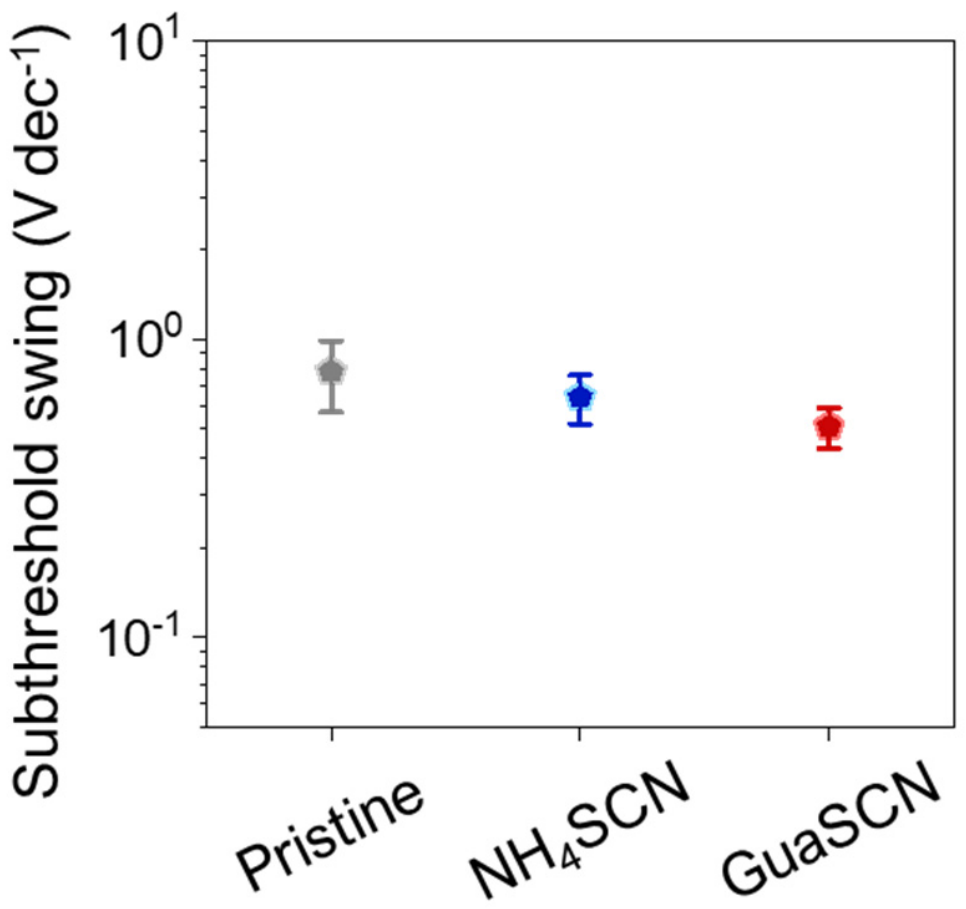

Fig. S14.

Subthreshold swing distribution of $FASnI_3$ TFTs. Subthreshold swing distribution of $FASnI_3$, NH_4SCN-treated, and GuaSCN-treated $FASnI_3$ TFT devices (mean and standard deviation from forward scan curves of 30 devices).

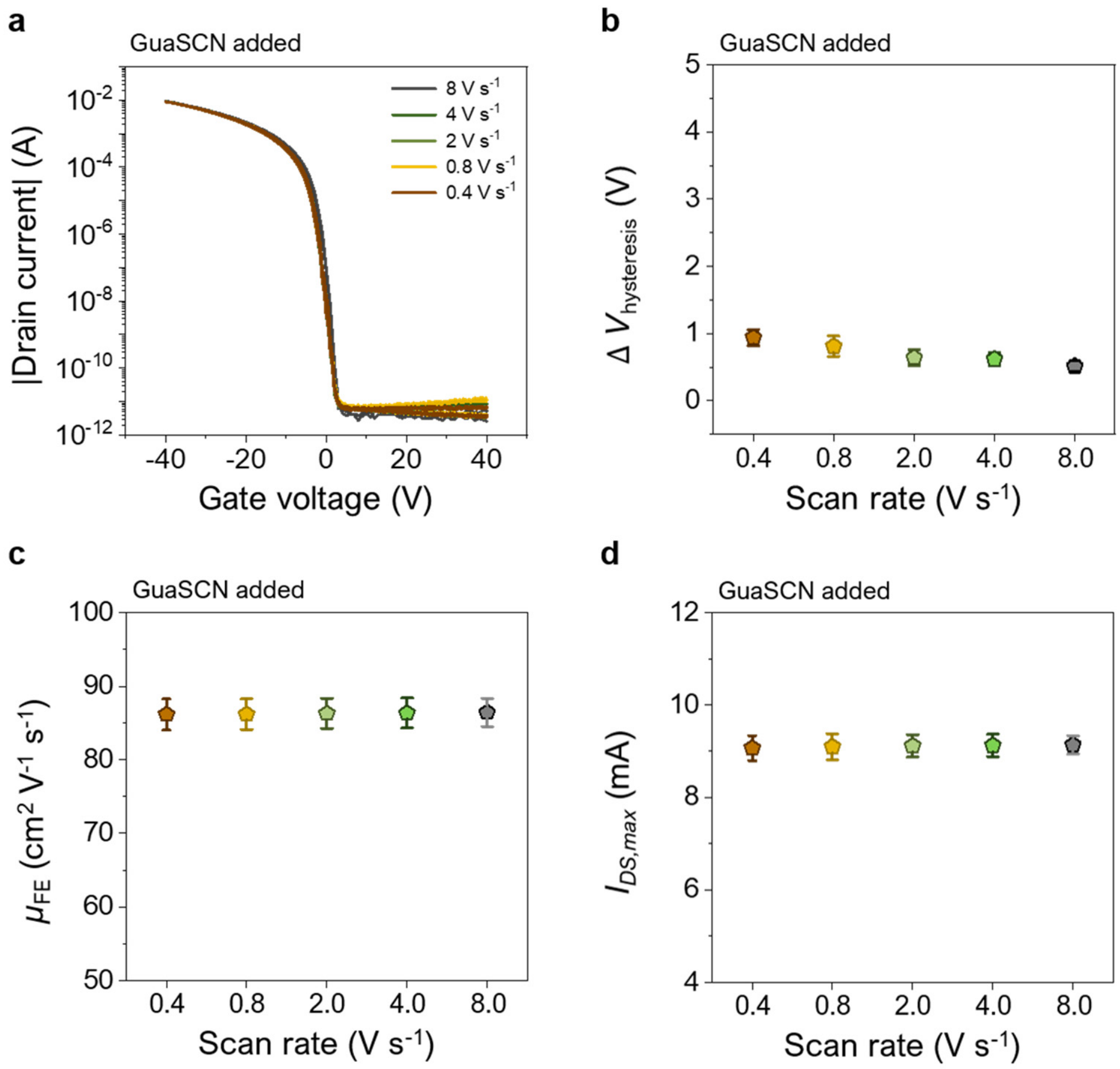

Fig. S15.

Sweep-rate-dependent hysteresis in GuaSCN-treated TFTs. (**A**) Dual-scan transfer characteristics of the GuaSCN-treated TFT measured at different gate-voltage sweep rates. (**B**) Hysteresis voltage ($\Delta V_{\mathrm{Hysteresis}}$) extracted from 30 GuaSCN-treated TFTs at each scan rate. (**C**) Field-effect mobility (μ_{FE}) and (**D**) maximum drain current ($I_{\mathrm{DS,max}}$) extracted at each gate-voltage sweep rate. Symbols and error bars represent the mean and standard deviation, respectively, from 30 devices at each condition.

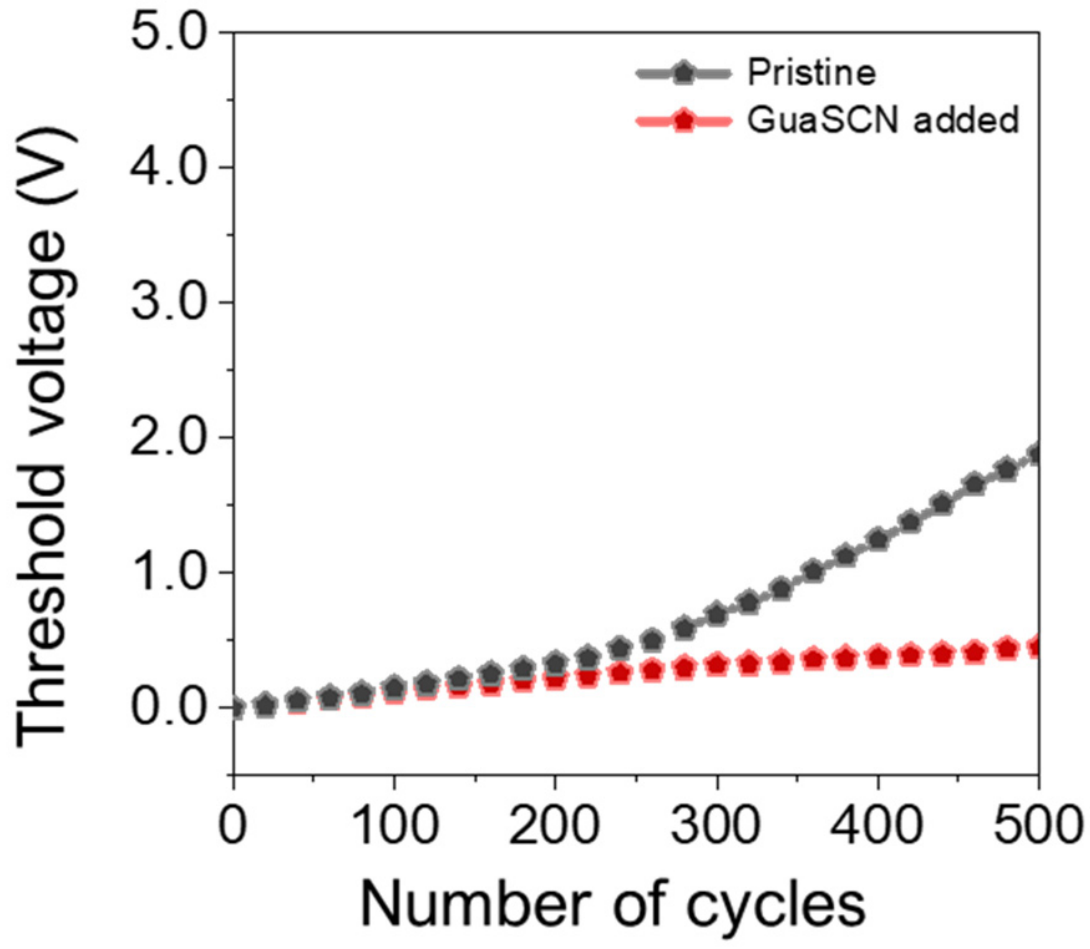

Fig. S16.
Threshold-voltage evolution. Threshold-voltage evolution during repeated switching cycles for pristine and GuaSCN-treated Sn perovskite TFTs.

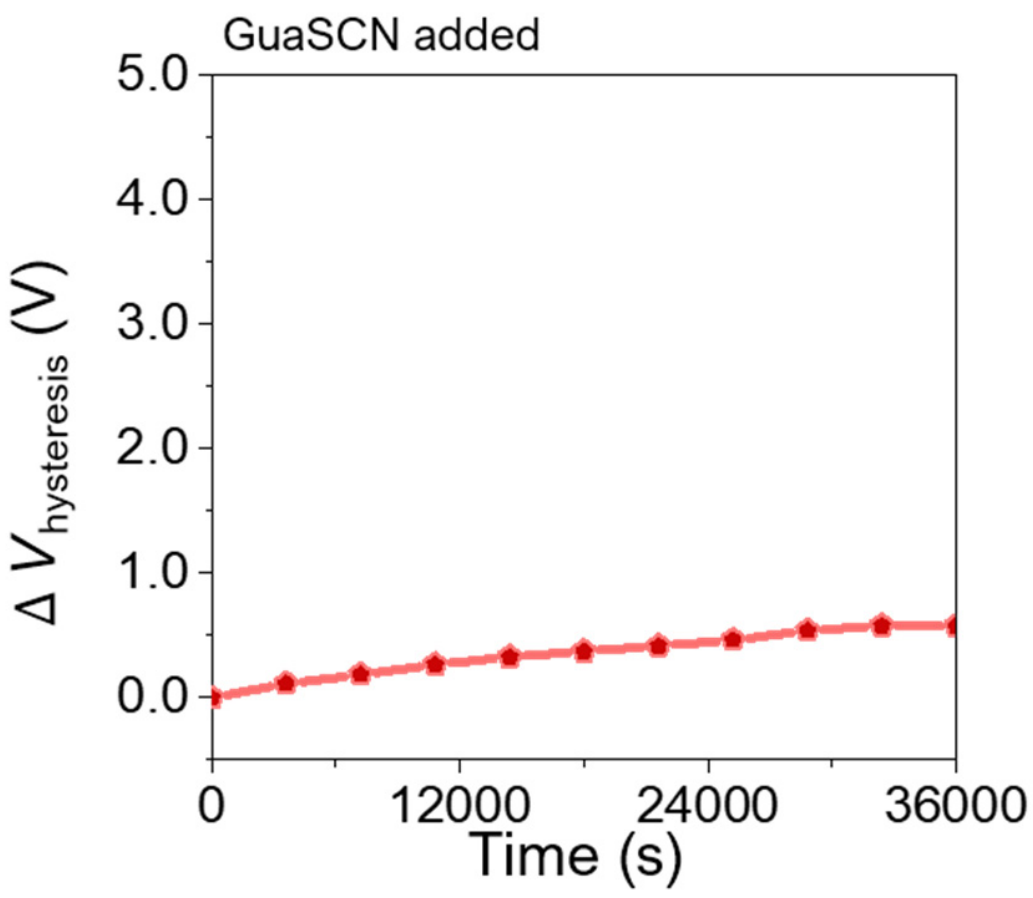

Fig. S17.

Hysteresis evolution. Hysteresis evolution of GuaSCN-treated TFTs under prolonged gate-bias stressing.

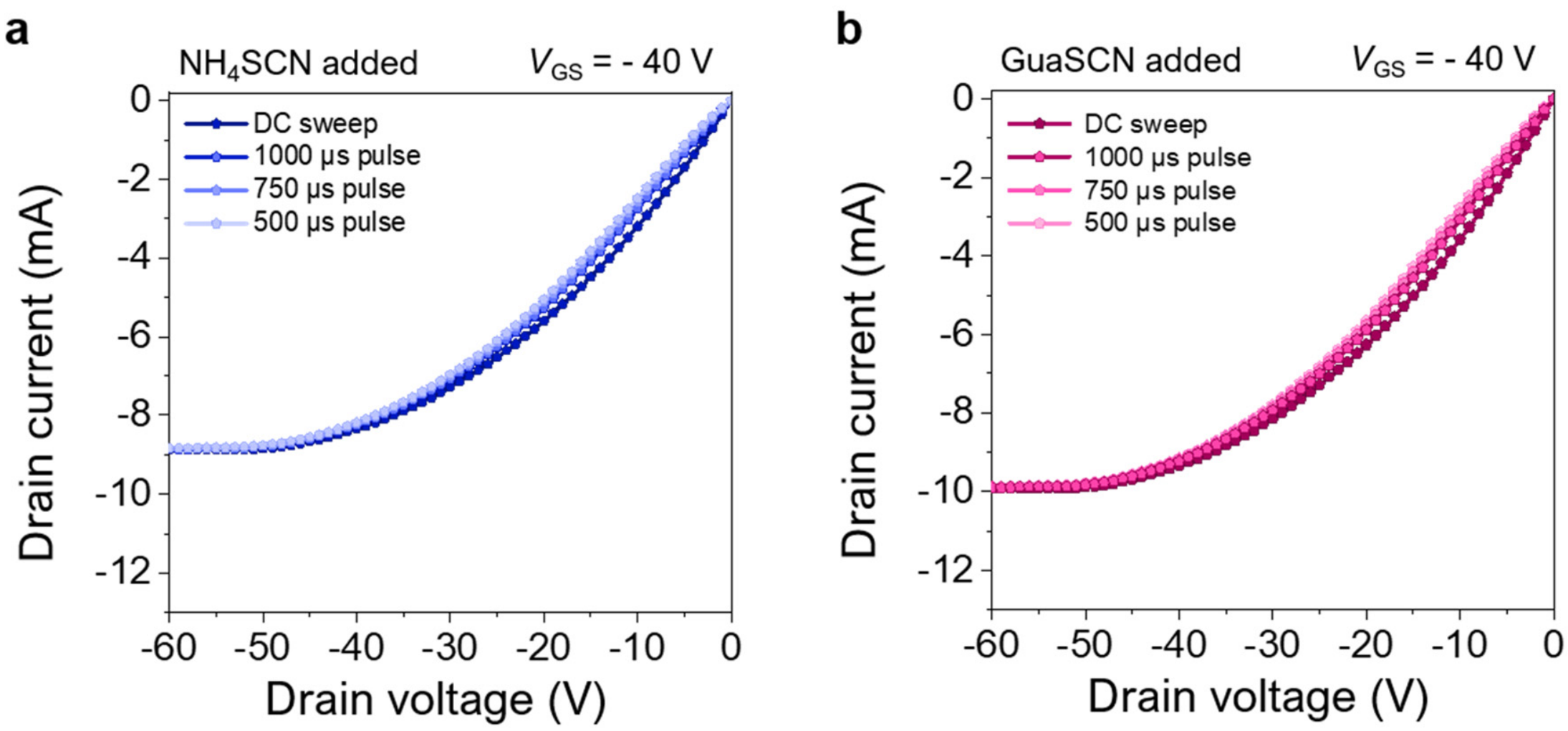

Fig. S18.

Pulse-width-dependent output characteristics of SCN-treated TFTs. Output characteristics of (**A**) NH_4SCN-treated and (**B**) GuaSCN-treated TFTs measured at V_{GS} = −40 V using conventional dc sweeping and drain-voltage pulses with widths of 1000, 750, and 500 µs. The pulsed output characteristics closely overlap with the corresponding dc-sweep characteristics, with a maximum deviation in drain current of approximately 1.8% at V_{DS} = −40 V. Panel (B) is reproduced in Fig. 3F.

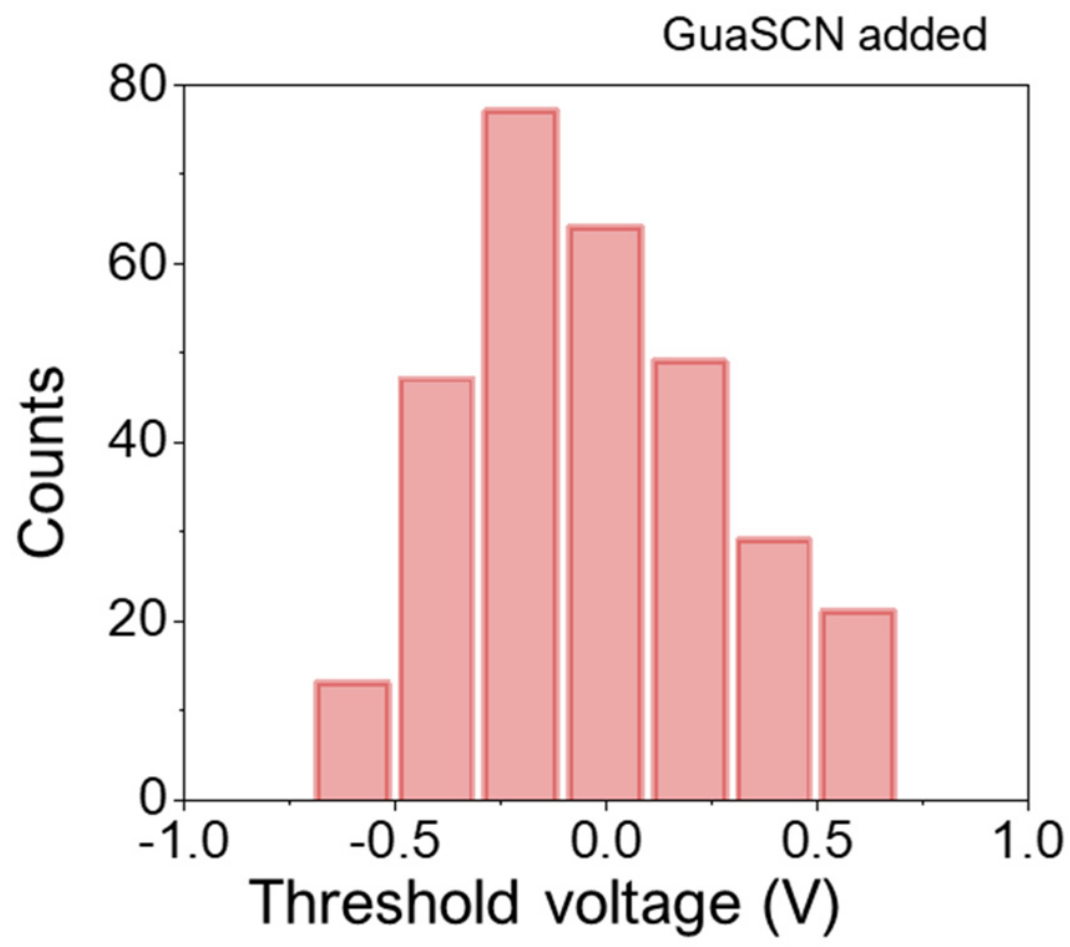

Fig. S19.
Threshold-voltage distribution and device uniformity. Histogram of threshold voltage values extracted from 300 GuaSCN-treated $FASnI_3$ TFTs. The narrow distribution, centered at −0.02 V with a standard deviation of 0.34 V, highlights the excellent device-to-device uniformity.

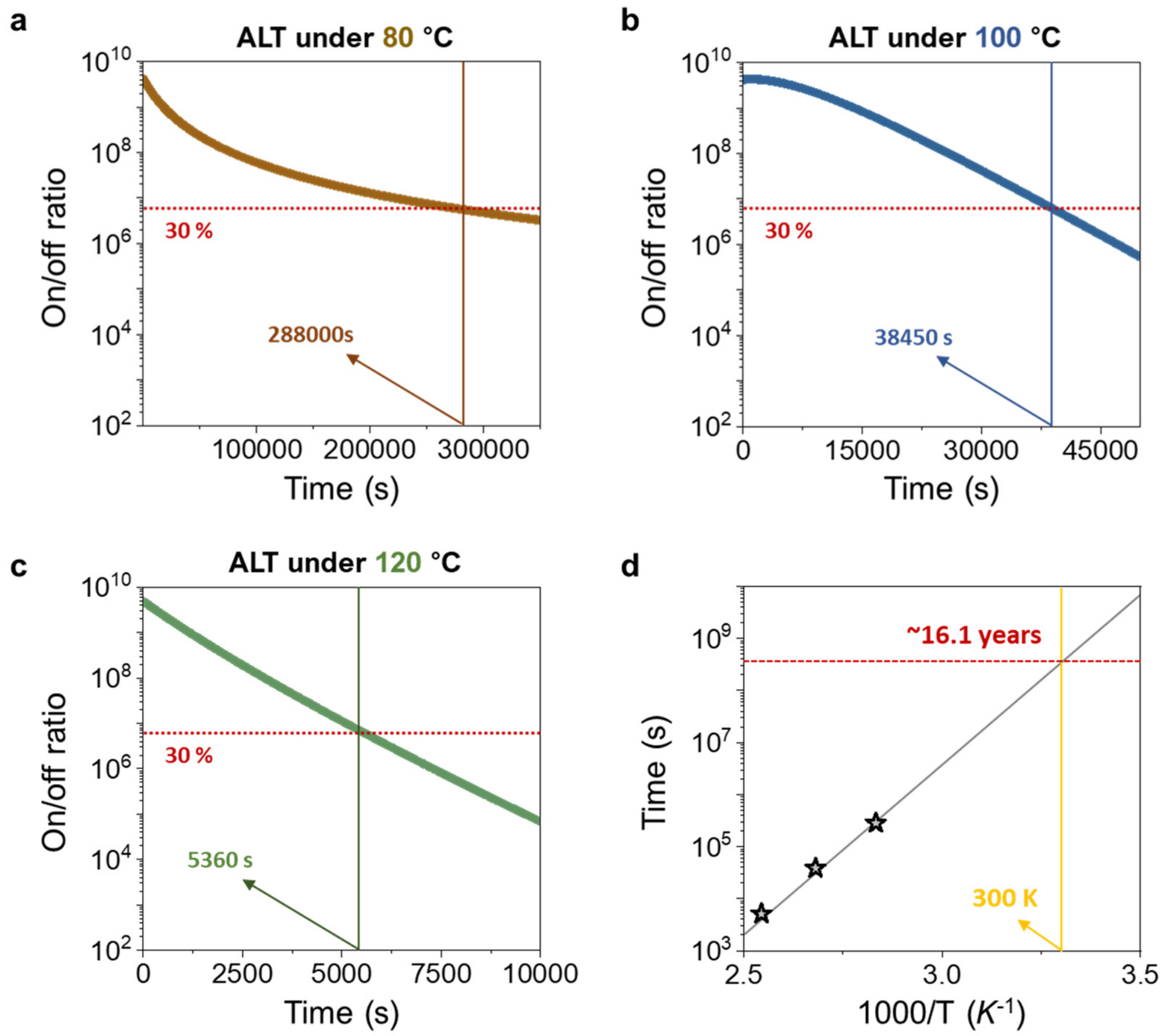

Fig. S20.

Operational lifetime estimation using accelerated aging tests. Variation of the on/off ratio of encapsulated GuaSCN-treated TFTs measured under accelerated aging conditions at (**A**) 80 °C, (**B**) 100 °C, and (**C**) 120 °C, respectively. The characteristic degradation lifetime was extracted using a 30% retention criterion (red dashed line). (**D**) Arrhenius plot of the extracted degradation lifetimes as a function of inverse temperature. Linear fitting based on the Arrhenius relation was used to extrapolate the operational lifetime at room temperature (300 K), yielding an estimated lifetime of ~16.1 years.

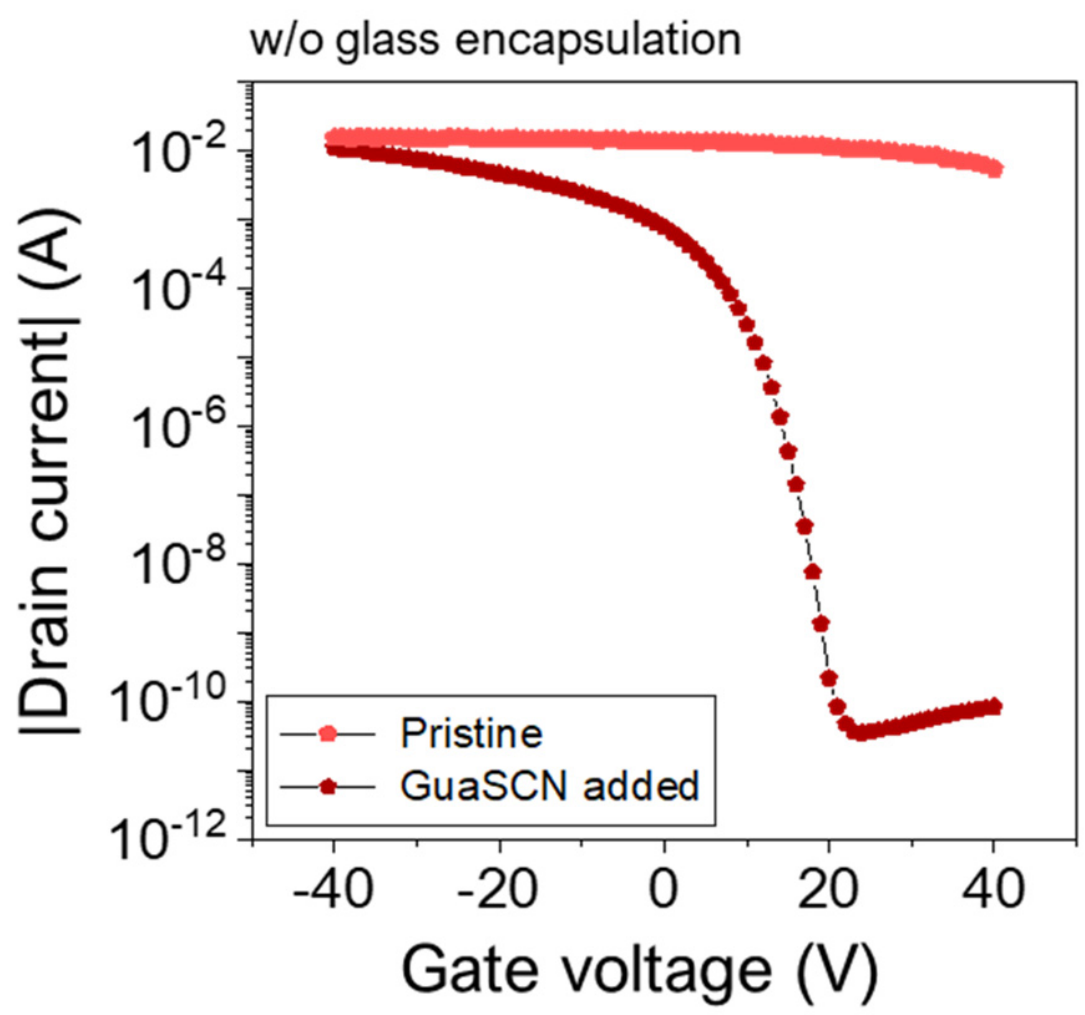

Fig. S21.

Air-exposure stability of pristine and GuaSCN-treated TFTs. Transfer characteristics of pristine and GuaSCN-treated TFTs measured after 5 min exposure to ambient air without encapsulation.

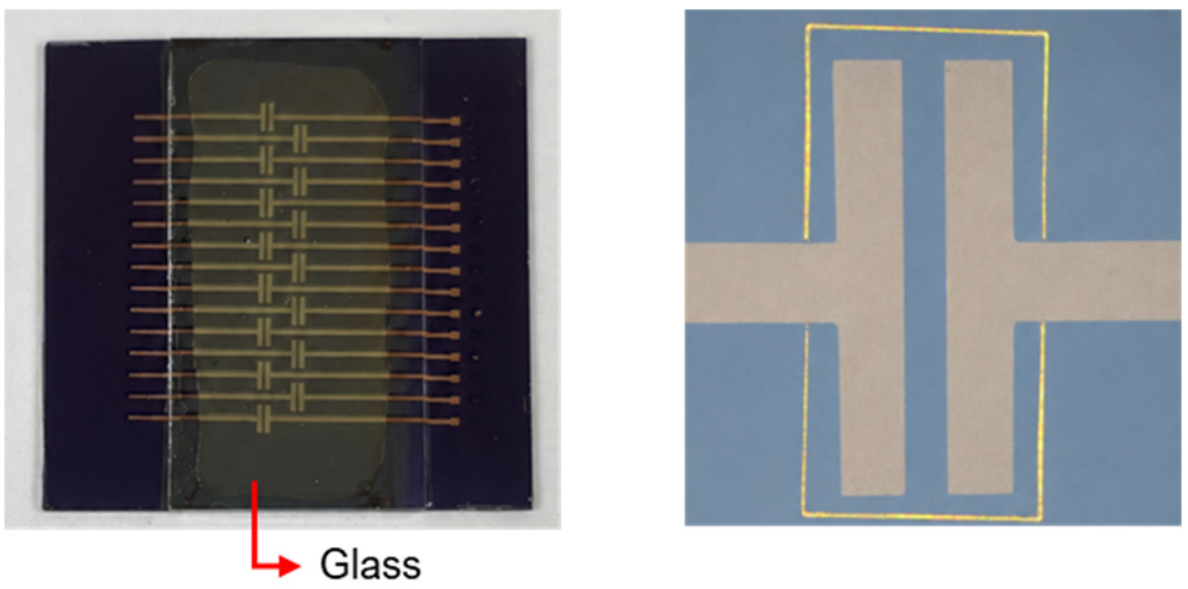

Fig. S22.

Optical photograph of the glass-encapsulated Sn perovskite TFT devices. Left: optical photograph of the fully encapsulated TFT devices. Right: enlarged optical image of the encapsulated device region.

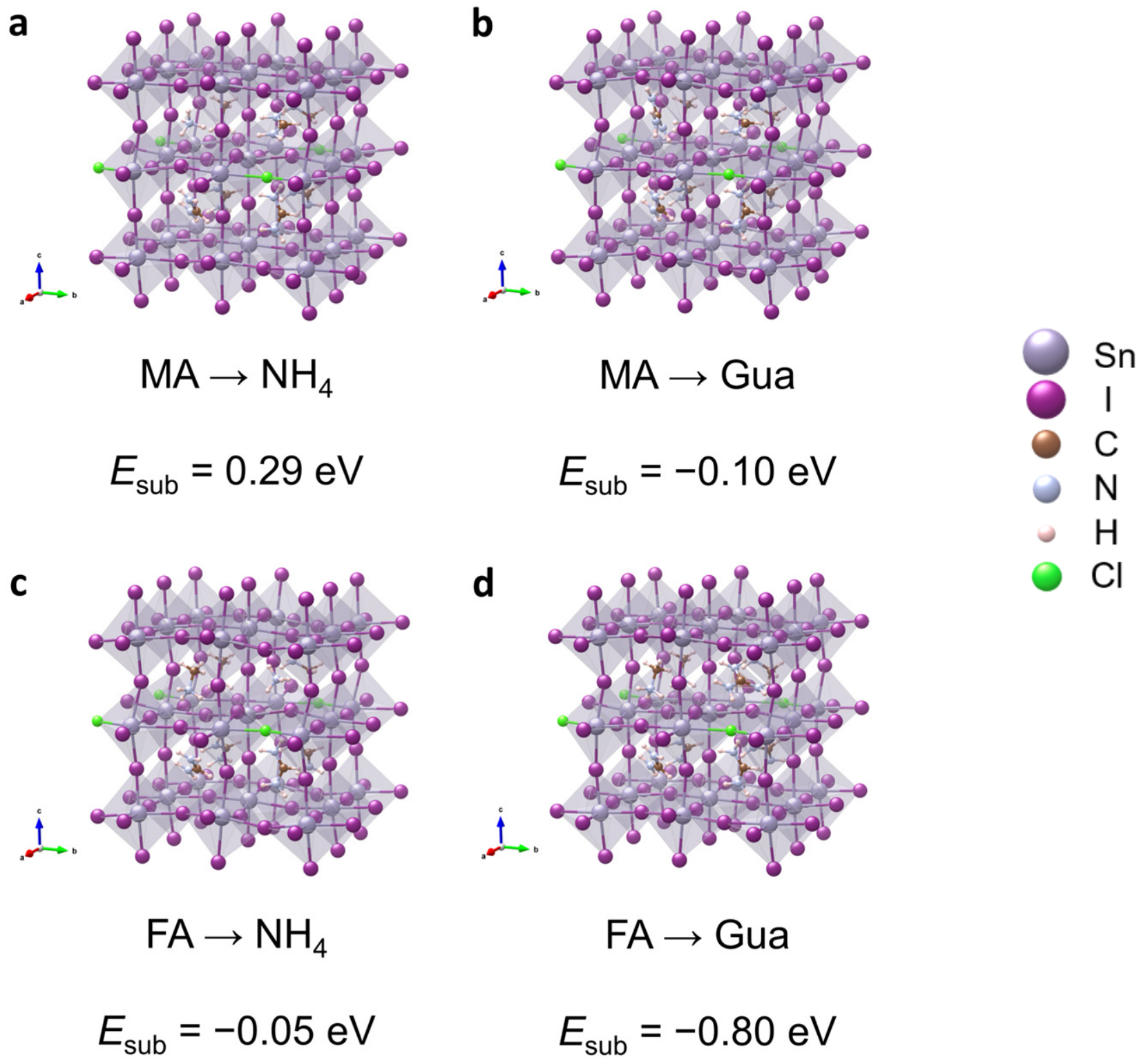

Fig. S23.
Preference of NH_4 and Gua substitution in mixed-cation perovskites. Substitutional energy (E_{sub}) at (**A**) MA to NH_4, (**B**) MA to Gua, (**C**) FA to NH_4, and (**D**) FA to Gua in $FA_{0.625}MA_{0.375}SnI_{2.875}Cl_{0.125}$.

Supplementary Note 1. Extraction and validation of field-effect mobility in Sn halide perovskite TFT devices.

Threshold voltage (V_T), apparent field-effect mobility (μ_{FE}), and reliability-corrected effective mobility ($\mu_{effective}$) were extracted from the forward (off-to-on) transfer characteristics measured at V_{DS} = -40 V in the saturation regime. The mobility extraction followed the conventional saturation-regime field-effect transistor (FET) model (*51*),

$$I_{DS,sat} = \frac{\mu_{FE,sat}\, W\, C_i}{2L}(V_{GS} - V_T)^2\ ,\ |V_{GS} - V_T| < |V_{DS}| \quad \text{(S1)}$$

from which $\mu_{FE,sat}$ is obtained from the slope of the $\sqrt{|I_{DS}|} - V_{GS}$ plot as

$$\mu_{FE,sat} = \frac{2L}{WC_i}\left(\frac{\partial\sqrt{|I_{DS}|}}{\partial V_{GS}}\right)^2 \quad \text{(S2)}$$

where L and W are the channel length and width, respectively, C_i is the gate-dielectric capacitance per unit area, and I_{DS} and V_{GS} denote the drain current and gate voltage. For all devices, L = 100 µm, W = 1500 µm, and the experimentally measured C_i = 9.82 nF cm^{-2} were used.

For extraction of the reported μ_{FE} values, $\sqrt{|I_{DS}|}$ was plotted as a function of V_{GS} and linearly fitted over the range -40 V ≤ V_{GS} ≤ -5 V (Fig. S11A). The corresponding derivative, $\partial\sqrt{|I_{DS}|}/\partial V_{GS}$, was evaluated over same gate-voltage range (yellow-shaded region in Fig. S11B) and converted into gate-voltage-dependent μ_{FE} values (Fig. S11C). The μ_{FE} obtained within this range were then averaged to determine the reported mobility for each device. The same fitting range and extraction procedure were consistently applied to all devices included in the statistical analysis (Table S3).

Figure S11A shows the forward- and reverse-sweep $\sqrt{|I_{DS}|}$–V_{GS} characteristics of the GuaSCN-treated TFT. Within the fitting range, $\partial\sqrt{|I_{DS}|}/\partial V_{GS}$ remains nearly constant (Fig. S11B), resulting in only weak V_{GS} dependence of the extracted μ_{FE} (Fig. S11C). This confirms that the reported mobility is derived from an extended linear region of the transfer characteristic rather than from a narrow, locally steep segment.

Because Sn halide perovskites exhibit mixed ionic–electronic transport, possible non-idealities associated with ionic redistribution and hysteresis were additionally examined. Forward and reverse transfer characteristics are compared in Figs. 3A,B, S10 and S11, while scan-rate-dependent dual-sweep measurements are presented in Fig. S15. Unless otherwise noted, the mobility values reported in the main text and statistical analyses were extracted from the forward sweeps.

To account for potential overestimation of the apparent mobility arising from non-ideal transfer characteristics, the measurement reliability factor (*r*) was evaluated according to the previously reported method (*40*). This factor is defined as the ratio of the maximum channel conductivity of an equivalent ideal transistor to the experimentally achieved channel conductivity. In the saturation regime, r_{sat} is defined as

$$r_{\mathrm{sat}} = \left(\frac{\sqrt{|I_{\mathrm{DS}}|^{max}} - \sqrt{|I_{\mathrm{DS}}^{0}|}}{|V_{\mathrm{GS}}|^{max}} \right)^{2} \Bigg/ \left(\frac{\partial \sqrt{|I_{\mathrm{DS}}|}}{\partial V_{\mathrm{GS}}} \right)^{2} \quad \text{(S3)}$$

where $|I_{\mathrm{DS}}|^{\mathrm{max}}$ is the drain current at $|V_{\mathrm{GS}}| = |V_{\mathrm{GS}}|^{\mathrm{max}}$ (here, 40 V), and $|I_{\mathrm{DS}}^{0}|$ is the current at $V_{GS} = 0$ V. The reliability-corrected effective mobility is then given by

$$\mu_{\mathrm{effective}} = r \times \mu_{\mathrm{FE}} \quad \text{(S4)}$$

For the champion GuaSCN-treated TFT, which exhibits μ_{FE} = 88.6 cm^2 V^{-1} s^{-1}, the measurement reliability factor is *r* = 0.971, corresponding to $\mu_{effective}$ = 86.0 cm^2 V^{-1} s^{-1}. This value is provided as a conservative estimate of the champion-device mobility. The corresponding statistical values of μ_{FE}, *r*, and $\mu_{effective}$ for pristine, NH_4SCN-treated, and GuaSCN-treated TFTs are summarized in Table S3.

Supplementary Note 2. Assessment of self-heating and non-equilibrium effects under high-current operation.

For the best-performing GuaSCN-treated TFT shown in Fig. 3B, which exhibited a field-effect mobility of 88.6 $cm^2\ V^{-1}\ s^{-1}$, the maximum drain current was 9.93 × 10^{-3} A at V_{DS} = -40 V. Using a channel width (W) of 1500 μm and channel length (L) of 100 μm, the maximum areal Joule power density was calculated as $P_{\max} = \frac{|I_{\mathrm{DS,max}} \times V_{\mathrm{DS}}|}{W \times L}$, yielding approximately 2.65 × 10^2 W cm^{-2}. The corresponding maximum source-drain current density was estimated as $j_{\mathrm{max,DS}} = \frac{\Delta I_{\mathrm{DS,max}}}{W \times t}$, where t = 3 nm is the nominal accumulation-channel thickness adopted to comparison with the previous estimations (*52*). This yields $j_{\mathrm{max,DS}} \approx 2.21 \times 10^5$ A cm^{-2}. We emphasize that this value represents an effective current-density estimate based on an assumed accumulation-layer thickness rather than a direct measurement of the depth-dependent current distribution within the perovskite channel.

These quantitative estimates place the devices in a high-power and high-current-density operating regime for which non-equilibrium effects warrant careful consideration. Importantly, however, the calculated power and current densities alone do not establish that self-heating or other non-equilibrium processes measurably perturb the electrical characteristics. We therefore performed complementary electrical analyses to determine whether such effects influence the reported mobility and drain current.

First, we have examined measurement-timescale-dependent changes in the transfer characteristics of the optimized GuaSCN-treated TFTs. The gate-voltage sweep rate was varied from 0.4 to 8 V s^{-1}, and the field-effect mobility and maximum drain current were extracted at each sweep rate (Fig. S15C,D). Neither parameter exhibited a systematic increase or decrease with sweep rate, and their distributions remained comparable throughout the investigated range. Consistently, the transfer characteristics and forward–reverse hysteresis showed only minor variations with sweep rate (Fig. S15A,B). These results indicate that the extracted mobility and on-state current are not measurably dependent on the transfer-measurement timescale over the investigated range.

Second, and more directly, we have newly assessed the possible influence of self-heating on the drain current by measuring the output characteristics using drain-voltage pulses with different pulse widths and comparing them with the conventional dc-sweep output characteristics for both NH_4SCN- and GuaSCN-treated TFTs (Fig. S18). Shorter drain-voltage pulses reduce the time available for thermal accumulation during the on state (*53*). Nevertheless, the output characteristics measured with pulse widths of 1,000 μs, 750 μs, and 500 μs closely overlapped with the corresponding dc-sweep curves for both devices, showing only minor variations in drain-current magnitude and overall curve

shape. At V_{GS} = -40 V and V_{DS} = -40 V, the drain currents of the NH_4SCN-treated TFT measured with pulse widths of 1,000 μs, 750 μs, and 500 μs deviated from the dc value by only 1.1%, 1.5%, and 1.8%, respectively. For the GuaSCN-treated TFT, including the condition corresponding to the highest-mobility device, the respective deviations were only 1.1%, 1.3%, and 1.4%, respectively. Thus, the maximum deviation from the dc-sweep current across both additive conditions was approximately 1.8%. Importantly, the drain current exhibited no systematic dependence on pulse width. Even when the electrical excitation duration was reduced by a factor of two, the drain current changed by no more than 1.8%, indicating that self-heating does not appreciably affect the measured output current within the investigated bias and pulse-width ranges. Although these electrical measurements do not directly probe the transient channel temperature, the negligible dependence of the drain current on pulse width provides experimental evidence that any transient self-heating, if present, has no measurable influence on the reported electrical characteristics under the investigated measurement conditions. The pulse width, pulse interval, and corresponding measurement conditions have now been specified in the revised Materials and Methods section.

Third, we examined whether repeated high-current electrical operation produced cumulative or irreversible device degradation. The GuaSCN-treated TFTs maintained nearly unchanged transfer characteristics during 500 consecutive forward–reverse sweeps, with no appreciable current degradation and only negligible variations in threshold voltage, on- and off-state currents, and subthreshold swing (Fig. 3H and Fig. S16). In addition, during prolonged gate-bias stressing for up to 36,000 s, the hysteresis voltage showed only a slight initial increase followed by gradual stabilization (Fig. 3I and Fig. S17). No discernible drift in either the on- or off-state current was observed over 12,000 on/off switching cycles (Fig. 3J). These stability measurements do not by themselves exclude a transient temperature rise during an individual electrical measurement; however, they demonstrate that repeated operation under the investigated bias conditions does not produce detectable cumulative electrical degradation.

Taken together, the quantitative power/current-density estimates, the absence of systematic sweep-rate dependence, the ≤ 1.8% deviation between the dc and pulsed output characteristics, and the highly reversible behavior during repeated electrical operation consistently indicate that the reported field-effect mobility and on-state current are robust against measurement-timescale-dependent non-equilibrium effects under the investigated conditions.

Table S1.
Fitted parameters extracted from time-resolved PL decay curves of $FASnI_3$ films without and with SCN-based additives, cast on quartz substrates (excitation wavelength: 635 nm).

	A_1(%)	τ_1(ns)	A_2(%)	τ_2(ns)	[a]τ_{avg}(ns)
**Pristine**	94.84	5.30	5.16	20.20	7.86
**NH_4SCN-treated**	64.87	5.35	35.13	15.53	11.57
**GuaSCN-treated**	81.45	8.71	18.55	26.95	16.25

The data are fitted with a biexponential function: $A_1 e^{-t/\tau_1} + A_2 e^{-t/\tau_2}$
[a]τ_{avg}: amplitude weighted average lifetime.

Table S2.
Device performance parameters of previously reported 3D (or 2D/3D mixture) Sn-based metal halide perovskite TFTs.

Composition / Dielectric, thickness (nm)	[a]Structure / Measured temperature	Channel W / L (μm)	Claimed field effect mobility ($cm^2V^{-1}s^{-1}$) / extraction method	$I_{on/off}$	SS ($V\ dec^{-1}$)	V_T (V)	Ref
**$FA_{0.85}MA_{0.15}SnI_{2.85}Cl_{0.15}$ / SiO_2, 300**	**BGTC / RT**	**1500 / 100**	**88.6 (max.), 86.5 (ave.) / saturation regime**	**4.4×10^9**	**0.49**	**-0.02**	**This work**
$MASn(I/Br/Cl)_3$ / HfO_2, 40	BGTC / RT	1000 / 150	19.6 / saturation regime	3.0×10^7	0.2	0	(*38*)
$CsSnI_3$ / SiO_2, 100	BGTC / RT	1000 / 150	28 / saturation regime	4.0×10^8	0.5	-2.8	(*54*)
$FA_{0.85}MA_{0.15}SnI_{2.85}Cl_{0.15}$ / SiO_2, 300	BGTC / RT	1500 / 100	82.5 (max.), 73.0 (ave.) / saturation regime	3.0×10^9	0.76	-0.01	(*30*)
$Cs_{0.15}FA_{0.85}SnI_3$ / SiO_2, 100	BGTC / RT	1200 / 150	74 / saturation regime	1.0×10^8	0.48	7	(*17*)
$(PEA)_2FA_6Sn_7I_{22}$ / SiO_2, 300	BGTC / RT	1000 / 100	53 / saturation regime	2.3×10^7	0.5	-2.4	(*55*)
$PEA_2FASn_2I_5SCN_2$ / SiO_2, 100	BGTC / RT	1200 / 150	43 / saturation regime	1.6×10^8	0.66	0.2	(*15*)
$CsSnI_2:PbCl_2$ / SiO_2, 100	BGTC / RT	750 / 550	33.8 / saturation regime	8.2×10^8	0.5	24	(*23*)
$FASnI_3$ / SiO_2, 100	BGTC / RT	1000 / 100	13.6 / saturation regime	8.9×10^7	0.32	32	(*56*)
$FASnI_3$ / SiO_2, 100	BGTC / RT	1000 / 100	40.7 / saturation regime	-	-	6.7	(*57*)
$CsSnI_2:PbCl_2$ / SiO_2, 300	BGTC / RT	1000 / 100	23.7 / saturation regime	2.1×10^7	-	-	(*58*)
$FASnI_3$ / SiO_2, 100	BGTC / RT	1000 / 100	28.6 / saturation regime	-	-	-	(*59*)
$4\text{-}FPEA_{0.17}FA_{0.83}SnI_3$ / HfO_2, 40	BGTC / RT	1000 / 100	11.8 / hall measurement	4.5×10^8	0.1	-	(*60*)
$PEA_{0.17}FA_{0.83}SnI_3$ / SiO_2, 230	BGTC / RT	10000 / 20	0.2 / linear regime	1.0×10^4	-	2.8	(*20*)
$PEA_{0.1}FA_{0.9}SnI_3$ / SiO_2, 150	BGTC / RT	1000 / 500	25.5 / linear regime	1.0×10^6	0.1	5.2	(*61*)
$PEA_{0.22}(Cs_xFA_{1-x})_{0.78}SnI_3$ / SiO_2, 100	BGTC / RT	1000 / 200	72 / saturation regime	2.6×10^8	0.5	20.4	(*22*)
$PEA_{2x}(FA_{0.9}Cs_{0.1})_{1-x}SnI_{(x+3)}$ / SiO_2, 100	BGTC / RT	1200 / 150	17.36 / saturation regime	6.8×10^7	0.75	33.4	(*62*)
$FA_{0.94}$(2D cation)$_{0.06}SnI_3$ / SiO_2, 100	BGTC / RT	1000 / 100	56.1 / saturation regime	1.0×10^7	-	-	(*63*)
$FPEA\text{-}FASnI_3$ / SiO_2, 100	BGTC / RT	1500 / 100	12.8 / saturation regime	7.8×10^7	0.1	2.4	(*21*)

$CsFASnI_3$ / SiO_2, 100	BGTC / RT	200 / 100	61 / saturation regime	3.0×10^8	0.5	18.7	(*64*)

[a] BGTC, TCTG and BGBC denote bottom-gate top-contact, top-contact top-gate and bottom-gate bottom-contact, respectively.

Table S3.
Statistical parameters extracted for $FASnI_3$ TFT devices without and with SCN-based additives (mean ± standard deviation from forward scan curves of 100 devices).

	[a] Claimed mobility, μ_{FE} [$cm^2 V^{-1} s^{-1}$]	Measurement reliability factor, r	[b] Effective mobility, $\mu_{effective}$ [$cm^2 V^{-1} s^{-1}$]	$I_{on/off}$ [$\times 10^9$]	Subthreshold Swing [$V\ dec^{-1}$]
**Pristine**	73.01 ± 8.03	0.96 ± 0.09	70.23 ± 11.28	1.02 ± 0.16	0.76 ± 0.02
**NH_4SCN-treated**	80.77 ± 4.11	0.96 ± 0.08	77.54 ± 7.68	2.75 ± 0.27	0.64 ± 0.12
**GuaSCN-treated**	86.51 ± 2.23	0.97 ± 0.07	84.01 ± 6.44	4.20 ± 0.16	0.51 ± 0.08

[a] Claimed mobility (μ_{FE}) values are extracted from the saturation regime (also denoted as $\mu_{FE,sat}$) (details are described in Supplementary Note 1)

[b] $\mu_{effective}$: effective mobility (= $r \times \mu_{FE}$)

REFERENCES

1. N. Park, Perovskite solar cells: An emerging photovoltaic technology. *Mater. Today* **18**, 65–72 (2015).

2. Z.-K. Tan, R. S. Moghaddam, M. L. Lai, P. Docampo, R. Higler, F. Deschler, M. Price, A. Sadhanala, L. M. Pazos, D. Credgington, F. Hanusch, T. Bein, H. J. Snaith, R. H. Friend, Bright light-emitting diodes based on organometal halide perovskite. *Nat. Nanotechnol.* **9**, 687–692 (2014).

3. H. Park, C. B. Lee, S.-J. Lim, H. Kim, K. Kim, H. J. Park, Near-infrared photodetection in tin halide perovskites via compositional engineering. *Mater. Sci. Eng. R Rep.* **165**, 101013 (2025).

4. L. Lei, Q. Dong, K. Gundogdu, F. So, Metal halide perovskites for laser applications. *Adv. Funct. Mater.* **31**, 2010144 (2021).

5. Y. Fu, H. Zhu, J. Chen, M. P. Hautzinger, X.-Y. Zhu, S. Jin, Metal halide perovskite nanostructures for optoelectronic applications and the study of physical properties. *Nat. Rev. Mater.* **4**, 169–188 (2019).

6. Y.-H. Chiang, M.-H. Li, H.-M. Cheng, P.-S. Shen, P. Chen, Mixed cation thiocyanate-based pseudohalide perovskite solar cells with high efficiency and stability. *ACS Appl. Mater. Interfaces* **9**, 2403–2409 (2017).

7. C. Wang, Z. Song, Y. Yu, D. Zhao, R. A. Awni, C. R. Grice, N. Shrestha, R. J. Ellingson, X. Zhao, Y. Yan, Synergistic effects of thiocyanate additive and cesium cations on improving the performance and initial illumination stability of efficient perovskite solar cells. *Sustain. Energy Fuels* **2**, 2435–2441 (2018).

8. Y.-M. Xie, X. Xu, C. Ma, M. Li, Y. Ma, C.-S. Lee, S.-W. Tsang, Synergistic effect of pseudo-halide thiocyanate anion and cesium cation on realizing high-performance pinhole-free MA-based wide-band gap perovskites. *ACS Appl. Mater. Interfaces* **11**, 25909–25916 (2019).

9. A. Mahapatra, D. Prochowicz, M. M. Tavakoli, S. Trivedi, P. Kumar, P. Yadav, A review of aspects of additive engineering in perovskite solar cells. *J. Mater. Chem. A* **8**, 27–54 (2020).

10. J. Hardy, H. Fiedler, J. Kennedy, A review on the current status and chemistry of tin halide perovskite films for photovoltaics. *Prog. Mater. Sci.* **151**, 101446 (2025).

11. H. Liu, Z. Zhang, F. Yang, J. Yang, A. N. Grace, J. Li, S. Tripathi, S. M. Jain, Dopants for enhanced performance of tin-based perovskite solar cells—A short review. *Coatings* **11**, 1045 (2021).

12. F. Gao, C. Li, L. Qin, L. Zhu, X. Huang, H. Liu, L. Liang, Y. Hou, Z. Lou, Y. Hu, F. Teng, Enhanced performance of tin halide perovskite solar cell by addition of lead thiocyanate. *RSC Adv.* **8**, 14025–14030 (2018).

13. D. Y. Heo, T. H. Lee, A. Iwan, L. Kavan, M. Omatova, E. Majkova, K. Kamarás, H. W. Jang, S. Y. Kim, Effect of lead thiocyanate ions on performance of tin-based perovskite solar cells. *J. Power Sources* **458**, 228067 (2020).

14. Z. Li, X. Li, M. Wang, M. Cai, X. Shi, Y. Mo, X. Chen, D. Ren, M. Yang, X. Liu, R. Jia, N. V. Medhekar, S. Dai, Enhanced photovoltaic performance via a bifunctional additive in tin-based perovskite solar cells. *ACS Appl. Energy Mater.* **5**, 108–115 (2022).

15. Y. Wu, S. Yang, E. Li, Y. Liu, F. Yuan, J. Wen, L. Hua, W. Wang, Y. Yang, Y. Lei, J. Chu, W. Li, Low-dimensional templates and delayed crystallization for high-quality tin-based perovskite films and high-performance transistors. *Nat. Commun.* **16**, 9505 (2025).

16. Z. Xie, M. Wei, X. Yang, W. Yin, J. Tian, C. Sheng, Z. Wang, Q.-S. Jiang, Synergistic enhancement of tin–lead perovskite solar cells using NH_4SCN and guanidinium-based additive for superior efficiency and stability. *ACS Appl. Mater. Interfaces* **17**, 62193–62204 (2025).

17. Y. Wu, F. Yuan, S. Yang, E. Li, W. Wang, Y. Liu, X. Yang, J. Wen, L. Hua, Y. Yang, Y. Lei, J. Chu, W. Li, Tailoring tin-based perovskite crystallization via large cations and pseudo-halide anions for high mobility and high stable transistors. *Sci. Adv.* **11**, eadv4138 (2025).

18. W. Ke, M. G. Kanatzidis, Prospects for low-toxicity lead-free perovskite solar cells. *Nat. Commun.* **10**, 965 (2019).

19. T. J. Macdonald, L. Lanzetta, X. Liang, D. Ding, S. A. Haque, Engineering stable lead-free tin halide perovskite solar cells: Lessons from materials chemistry. *Adv. Mater.* **35**, e2206684 (2023).

20. S. Shao, W. Talsma, M. Pitaro, J. Dong, S. Kahmann, A. J. Rommens, G. Portale, M. A. Loi, Field-effect transistors based on formamidinium tin triiodide perovskite. *Adv. Funct. Mater.* **31**, 2008478 (2021).

21. Z. Zhou, Q. Li, M. Chen, X. Zheng, X. Wu, X. Lu, S. Tao, N. Zhao, High-mobility and bias-stable field-effect transistors based on lead-free formamidinium tin iodide perovskites. *ACS Energy Lett.* **8**, 4496–4505 (2023).

22. H. Zhu, W. Yang, Y. Reo, G. Zheng, S. Bai, A. Liu, Y.-Y. Noh, Tin perovskite transistors and complementary circuits based on A-site cation engineering. *Nat. Electron.* **6**, 650–657 (2023).

23. Y. Reo, T. Zou, T. Choi, S. Kim, J.-Y. Go, T. Roh, H. Ryu, Y.-S. Kim, A. Liu, H. Zhu, Y.-Y. Noh, Vapour-deposited high-performance tin perovskite transistors. *Nat. Electron.* **8**, 403–410 (2025).

24. A. Liu, H. Zhu, S. Bai, Y. Reo, M. Caironi, A. Petrozza, L. Dou, Y.-Y. Noh, High-performance metal halide perovskite transistors. *Nat. Electron.* **6**, 559–571 (2023).

25. M. P. Hautzinger, W. Mihalyi-Koch, S. Jin, A-site cation chemistry in halide perovskites. *Chem. Mater.* **36**, 10408–10420 (2024).

26. W. Li, M. Hao, A. Baktash, L. Wang, J. Etheridge, The role of ion migration, octahedral tilt, and the A-site cation on the instability of $Cs_{1-x}FA_xPbI_3$. *Nat. Commun.* **14**, 8523 (2023).

27. D. W. Ferdani, S. R. Pering, D. Ghosh, P. Kubiak, A. B. Walker, S. E. Lewis, A. L. Johnson, P. J. Baker, M. S. Islam, P. J. Cameron, Partial cation substitution reduces iodide ion transport in lead iodide perovskite solar cells. *Energy. Environ. Sci.* **12**, 2264–2272 (2019).

28. S. Tan, I. Yavuz, N. De Marco, T. Huang, S.-J. Lee, C. S. Choi, M. Wang, S. Nuryyeva, R. Wang, Y. Zhao, H.-C. Wang, T.-H. Han, B. Dunn, Y. Huang, J.-W. Lee, Y. Yang, Steric

impediment of ion migration contributes to improved operational stability of perovskite solar cells. *Adv. Mater.* **32**, e1906995 (2020).

29. N. K. Tailor, A. Mahapatra, A. Kalam, M. Pandey, P. Yadav, S. Satapathi, Influence of the A-site cation on hysteresis and ion migration in lead-free perovskite single crystals. *Phys. Rev. Mater.* **6**, 045401 (2022).

30. H. Park, C. B. Lee, J. Lee, S.-J. Lim, B. H. Jeong, H. Kim, S. J. Lee, H. Oh, H. Ahn, D. H. Kim, K. Kim, H. J. Park, Non-volatile methylammonium chloride substitution for tin halide perovskite transistors. *Nat. Electron.* **8**, 934–948 (2025).

31. F. Hao, C. C. Stoumpos, D. H. Cao, R. P. H. Chang, M. G. Kanatzidis, Lead-free solid-state organic–inorganic halide perovskite solar cells. *Nat. Photonics* **8**, 489–494 (2014).

32. N. D. Pham, V. T. Tiong, D. Yao, W. Martens, A. Guerrero, J. Bisquert, H. Wang, Guanidinium thiocyanate selective Ostwald ripening induced large grain for high performance perovskite solar cells. *Nano Energy* **41**, 476–487 (2017).

33. T. Wang, H.-L. Loi, J. Cao, Z. Qin, Z. Guan, Y. Xu, H. Cheng, M. G. Li, C.-S. Lee, X. Lu, F. Yan, High open circuit voltage over 1 V achieved in tin-based perovskite solar cells with a 2D/3D vertical heterojunction. *Adv. Sci.* **9**, e2200242 (2022).

34. Y. Yang, Y. Yan, M. Yang, S. Choi, K. Zhu, J. M. Luther, M. C. Beard, Low surface recombination velocity in solution-grown $CH_3NH_3PbBr_3$ perovskite single crystal. *Nat. Commun.* **6**, 7961 (2015).

35. J. Wu, S.-C. Liu, Z. Li, S. Wang, D.-J. Xue, Y. Lin, J.-S. Hu, Strain in perovskite solar cells: Origins, impacts and regulation. *Natl. Sci. Rev.* **8**, nwab047 (2021).

36. Y. Dong, W.-X. Zhu, D.-T. Wu, X. Li, R. J. E. Westbrook, C.-J. Huang, Z. Min, W. Hong, B. Wang, G. Min, S. Sathasivam, M. Palma, S. Dimitrov, C.-T. Lin, T. J. Macdonald, Crystal growth modulation of tin–lead halide perovskites via chaotropic agent. *J. Am. Chem. Soc.* **147**, 31578–31590 (2025).

37. H. Kim, Y. H. Lee, T. Lyu, J. H. Yoo, T. Park, J. H. Oh, Boosting the performance and stability of quasi-two-dimensional tin-based perovskite solar cells using the formamidinium thiocyanate additive. *J. Mater. Chem. A* **6**, 18173–18182 (2018).

38. H. Zhu, A. Liu, K. I. Shim, H. Jung, T. Zou, Y. Reo, H. Kim, J. W. Han, Y. Chen, H. Y. Chu, J. H. Lim, H.-J. Kim, S. Bai, Y.-Y. Noh, High-performance hysteresis-free perovskite transistors through anion engineering. *Nat. Commun.* **13**, 1741 (2022).

39. C. Tyznik, M. Waldrip, R. P. Sullivan, M. Mirhosseini, A. Berry, S. Dwyer, H. Y. Cakir, T. Coffey, Y.-L. Loo, O. D. Jurchescu, A study on contact resistance as a function of surface treatment in perovskite field-effect transistors. *ACS Appl. Electron. Mater.* **5**, 5343–5351 (2023).

40. H. H. Choi, K. Cho, C. D. Frisbie, H. Sirringhaus, V. Podzorov, Critical assessment of charge mobility extraction in FETs. *Nat. Mater.* **17**, 2–7 (2018).

41. B. H. Jeong, H. Kim, C. B. Lee, H. Park, J. Kim, K. Kim, H. J. Park, Template-assisted crystallization for tin halide perovskite transistors. *Nano Energy* **148**, 111621 (2026).

42. R. Lin, K. Xiao, Z. Qin, Q. Han, C. Zhang, M. Wei, M. I. Saidaminov, Y. Gao, J. Xu, M. Xiao, A. Li, J. Zhu, E. H. Sargent, H. Tan, Monolithic all-perovskite tandem solar cells with 24.8% efficiency exploiting comproportionation to suppress Sn(II) oxidation in precursor ink. *Nat. Energy* **4**, 864–873 (2019).

43. G. Kresse, J. Furthmüller, Efficient iterative schemes for *ab initio* total-energy calculations using a plane-wave basis set. *Phys. Rev. B* **54**, 11169–11186 (1996).

44. J. P. Perdew, K. Burke, M. Ernzerhof, Generalized gradient approximation made simple. *Phys. Rev. Lett.* **77**, 3865–3868 (1996).

45. H. J. Monkhorst, J. D. Pack, Special points for Brillouin-zone integrations. *Phys. Rev. B* **13**, 5188–5192 (1976).

46. S. Grimme, J. Antony, S. Ehrlich, H. Krieg, A consistent and accurate *ab initio* parametrization of density functional dispersion correction (DFT-D) for the 94 elements H-Pu. *J. Chem. Phys.* **132**, 154104 (2010).

47. S. Grimme, S. Ehrlich, L. Goerigk, Effect of the damping function in dispersion corrected density functional theory. *J. Comput. Chem.* **32**, 1456–1465 (2011).

48. R. Dronskowski, P. E. Bloechl, Crystal orbital Hamilton populations (COHP): Energy-resolved visualization of chemical bonding in solids based on density-functional calculations. *J. Phys. Chem.* **97**, 8617–8624 (1993).

49. G. Henkelman, B. P. Uberuaga, H. Jónsson, A climbing image nudged elastic band method for finding saddle points and minimum energy paths. *J. Chem. Phys.* **113**, 9901–9904 (2000).

50. P. Virtanen, R. Gommers, T. E. Oliphant, M. Haberland, T. Reddy, D. Cournapeau, E. Burovski, P. Peterson, W. Weckesser, J. Bright, S. J. van der Walt, M. Brett, J. Wilson, K. J. Millman, N. Mayorov, A. R. J. Nelson, E. Jones, R. Kern, E. Larson, C. J. Carey, İ. Polat, Y. Feng, E. W. Moore, J. VanderPlas, D. Laxalde, J. Perktold, R. Cimrman, I. Henriksen, E. A. Quintero, C. R. Harris, A. M. Archibald, A. H. Ribeiro, F. Pedregosa, P. van Mulbregt, SciPy 1.0 Contributors, SciPy 1.0: Fundamental algorithms for scientific computing in Python. *Nat. Methods* **17**, 261–272 (2020).

51. S. M. Sze, K. K. Ng, *Physics of Semiconductor Devices*, (John Wiley & Sons, Hoboken, NJ, ed. 3, 2007).

52. V. Podzorov, V. Bruevich, Safe practices for mobility evaluation in field-effect transistors and Hall effect measurements using emerging materials. *Nat. Electron.* **7**, 266–268 (2024).

53. X. Li, L. Yang, S. Li, M. Huang, P. Ye, Y. Wu, Performance potential and limit of MoS_2 transistors. *Adv. Mater.* **27**, 1547–1552 (2015).

54. A. Liu, H. Zhu, S. Kim, Y. Reo, Y.-S. Kim, S. Bai, Y.-Y. Noh, Antimony fluoride (SbF_3): A potent hole suppressor for tin(II)-halide perovskite devices. *InfoMat* **5**, e12386 (2023).

55. J.-H. Kim, C.-M. Oh, I.-W. Hwang, K. Park, K. Lee, Organic surface doping for high-performance perovskite transistors. *Adv. Funct. Mater.* **35**, 2411836 (2025).

56. W. Yang, W. Park, Y. Reo, S. Yoo, S. Cho, H. Kim, S. Ryu, Y.-Y. Noh, Additive-free thermally evaporated tin halide perovskite transistors. *Mater. Sci. Eng. R Rep.* **168**, 101141 (2026).

57. K. Zhang, X. Wang, X. Zhang, H. Wang, W. Zheng, L. Zhang, High-performance tin-based perovskite field-effect transistors realized by improving film morphology via bifunctional additives. *Sci. Bull.* **71**, 89–97 (2026).

58. T. Roh, Y. Reo, S. Heo, G. Park, W. Yang, B. Ham, J. An, J.-H. Jung, S.-H. Baek, R.-H. Baek, C.-J. Kim, Y.-Y. Noh, Growth of tin halide perovskite film on two-dimensional hexagonal boron nitride via thermal evaporation. *ACS Energy Lett.* **10**, 5084–5092 (2025).

59. K. Zhang, W. Yang, H. Wang, Regulating growth kinetics of tin-based perovskites via ionic liquid additives for high-performance field-effect transistors. *Small* **21**, e08031 (2025).

60. W. Yang, G. Park, A. Liu, H. B. Lee, J.-W. Kang, H. Zhu, Y.-Y. Noh, Fluorinated organic A-cation enabling high-performance hysteresis-free 2D/3D hybrid tin perovskite transistors. *Adv. Funct. Mater.* **33**, 2303309 (2023).

61. J. Kim, Y.-S. Shiah, K. Sim, S. Iimura, K. Abe, M. Tsuji, M. Sasase, H. Hosono, High-performance p-channel tin halide perovskite thin film transistor utilizing a 2D–3D core–shell structure. *Adv. Sci.* **9**, e2104993 (2022).

62. X. Yang, Y. Liu, S. Yang, Y. Wu, Y. Lei, Y. Yang, A. Liu, J. Chu, W. Li, High-performance tin-halide perovskite transistors enabled by multiple A-cation engineering. *Adv. Funct. Mater.* **34**, 2403917 (2024).

63. W. Yang, K. Zhang, W. Yuan, L. Zhang, C. Qin, H. Wang, Enhancing stability and performance in tin-based perovskite field-effect transistors through hydrogen bond suppression of organic cation migration. *Adv. Mater.* **36**, e2313461 (2024).

64. G. Park, W. Yang, A. Liu, H. Zhu, F. De Angelis, Y.-Y. Noh, High-performance tin perovskite transistors through formate pseudohalide engineering. *Mater. Sci. Eng. R Rep.* **159**, 100806 (2024).
